# Attribution of Illnesses Transmitted by Food and Water to Comprehensive Transmission Pathways Using Structured Expert Judgment, United States

**DOI:** 10.3201/eid2701.200316

**Published:** 2021-01

**Authors:** Elizabeth Beshearse, Beau B. Bruce, Gabriela F. Nane, Roger M. Cooke, Willy Aspinall, Tine Hald, Stacy M. Crim, Patricia M. Griffin, Kathleen E. Fullerton, Sarah A. Collier, Katharine M. Benedict, Michael J. Beach, Aron J. Hall, Arie H. Havelaar

**Affiliations:** University of Florida, Gainesville, Florida, USA (E. Beshearse, A.H. Havelaar);; Centers for Disease Control and Prevention, Atlanta, Georgia, USA (B.B. Bruce, S.M. Crim, P.M. Griffin, K.E. Fullerton, S.A. Collier, K.M. Benedict, M.J. Beach, A.J. Hall);; Delft University of Technology, Delft, the Netherlands (G.F. Nane);; Resources for the Future, Washington, DC, USA (R. Cooke);; Aspinall & Associates, Tisbury, UK (W. Aspinall);; University of Bristol, Bristol, UK (W. Aspinall); Technical University of Denmark, Lyngby, Denmark (T. Hald)

**Keywords:** foodborne diseases, waterborne diseases, parasitic diseases, bacterial diseases, viral diseases, viruses, bacteria, parasites, pathway attribution, structured expert judgment, United States

## Abstract

Illnesses transmitted by food and water cause a major disease burden in the United States despite advancements in food safety, water treatment, and sanitation. We report estimates from a structured expert judgment study using 48 experts who applied Cooke’s classical model of the proportion of disease attributable to 5 major transmission pathways (foodborne, waterborne, person-to-person, animal contact, and environmental) and 6 subpathways (food handler–related, under foodborne; recreational, drinking, and nonrecreational/nondrinking, under waterborne; and presumed person-to-person-associated and presumed animal contact-associated, under environmental). Estimates for 33 pathogens were elicited, including bacteria such as *Salmonella enterica, Campylobacter* spp., *Legionella* spp., and *Pseudomonas* spp.; protozoa such as *Acanthamoeba* spp., *Cyclospora cayetanensis*, and *Naegleria fowleri*; and viruses such as norovirus, rotavirus, and hepatitis A virus. The results highlight the importance of multiple pathways in the transmission of the included pathogens and can be used to guide prioritization of public health interventions.

Illnesses transmitted commonly by food and water result in a major disease burden on both a national and a global scale (*1*). Each year in the United States, ≈9.4 million illnesses, 56,000 hospitalizations, and 1,351 deaths are caused by 31 known pathogens transmitted through food (*2*). Previous estimates of the burden of waterborne disease in the United States have largely focused on the burden of gastrointestinal illness associated with drinking water; an estimated 4–32 million cases of illness occur each year (*3*,*4*).

Source attribution is a process of estimating the proportion of illnesses resulting from various exposures for specific pathogens. Attributing illnesses to sources can guide decisions about where to target prevention and control efforts by apportioning illnesses to specific sources, thus aiding in the development of specific interventions (*5*). Attributing to the comprehensive set of transmission pathways considered in this study (foodborne, waterborne, person-to-person, animal contact, and environmental) is challenging for many reasons, including limited data and difficulty combining existing data from multiple sources. For example, outbreak surveillance data, such as those collected through the National Outbreak Reporting System (NORS), can provide information on sources of illness but are subject to reporting biases and may not be representative of endemic disease (*6*). Other studies have also raised concerns of publication bias toward novel, unique, or large foodborne outbreaks, limiting the utility of systematic reviews of published outbreaks in assessing source attribution (*7*,*8*). One method to address these barriers is structured expert judgment (SEJ), a method to use and combine estimates produced by experts and quantify uncertainty for the purpose of risk analysis when the ability to gather data is hindered by high expense, data scarcity, or lack of reliable data. This method, when executed well, is formal, reproducible, and mathematically and scientifically rigorous (*9*–*11*).

The Centers for Disease Control and Prevention (CDC) works to control and prevent illness caused by foodborne and waterborne pathogens in the United States. To accomplish this, CDC supports states and territories in tracking disease, detects and responds to outbreaks, and uses surveillance and sentinel site data to estimate the burden of these diseases in the United States. To inform this work, we implemented an SEJ study using Cooke’s classical model to estimate the proportion of domestically acquired illnesses for 33 pathogens transmitted through food and water that can be attributed to each of 5 major transmission pathways and 6 subpathways (*12*).

## Methods

The process was divided into 3 stages: preparation, elicitation, and postelicitation (*11*). These stages are detailed in the following sections.

### Preparation

#### Selection of Pathogens

We included all pathogens transmitted commonly through food or water that were examined by Scallan et al. (*2*) and Collier et al. (*13*) except those for which the only syndrome of interest was considered to have >95% foodborne transmission (e.g., *Listeria monocytogenes*, *Clostridium botulinum*); we added 3 free-living amoebae (*2*,*13*). For some pathogens, subdivisions into categories by serotype, patient age, or clinical manifestations of interest were included because transmission pathways were assumed to be different. For example, for *Salmonella*, the 5 most common serotypes were included along with 2 groups of rarer serotypes based on a ranking of their coefficients of variation (CVs) calculated from the patients’ ages, sexes, states of residence, and the year and month specimens were obtained (group 1, lowest CVs; group 2, highest CVs) as described by Boore et al. (*14*). This compilation resulted in a total of 33 pathogens and 47 target questions, or categories, for estimation. The 47 target questions were grouped into 15 panels on the basis of similarities between pathogen microbiology and ecology (Table 1).

**Table 1:** Pathogen panels, target questions, and number of experts providing estimates, structured expert judgment, United States, 2017

Panel	Pathogen and clinical manifestation target questions	No. experts who provided estimates in initial elicitation	No. experts who revised estimates	No. experts who provided re-elicitation estimates
Panel 1	*Acanthamoeba* spp., *Balamuthia mandrillaris, Naegleria fowleri*	14	4	Not required
Panel 2	Astrovirus, norovirus, rotavirus, sapovirus	17	3	Not required
Panel 3	*Brucella* spp., *Mycobacterium bovis*	16	5	Not required
Panel 4	*Campylobacter* spp., *Yersinia enterocolitica*	19	5	Not required
Panel 5	*Cryptosporidium* spp.,*Giardia* spp.	21	5	Not required
Panel 6	*Cyclospora cayetanensis*	21	4	Not required
Panel 7	Enterotoxigenic *Escherichia coli,* other diarrheagenic *E. coli, Shigella* spp.	21	3	Not required
Panel 8	Hepatitis A virus	19	2	Not required
Panel 9	*Legionella* spp., nontuberculous *Mycobacterium* spp.	9	1	Not required
Panel 10	*Pseudomonas* spp., otitis externa, pneumonia, septicemia	16	7	7
Panel 11	*Salmonella enterica*, nontyphoidal: all serotypes and ages, <5 y of age; Enteritidis, Typhimurium, Newport, I 4,[5],12:i:-, Javiana; other serotypes group 1,* other serotypes group 2†	14	3	Not required
Panel 12	Shiga toxin–producing *E. coli* O157 and non-O157	18	4	Not required
Panel 13	*Staphylococcus aureus*, group A *Streptococcus*	19	4	Not required
Panel 14	*Toxoplasma gondii*	16	3	Not required
Panel 15	*Vibrio alginolyticus*, AGI, non-AGI; *V. cholerae*, nontoxigenic, AGI, non-AGI; *V. parahaemolyticus*, AGI, non-AGI; *V. vulnificus*,‡ non-AGI; *Vibrio* spp., other, AGI, non-AGI	15	6	9

*Group 1: serotypes such as Agona, Anatum, Braenderup, Hadar, Heidelberg, Infantis, Oranienburg, Saintpaul, Senftenberg, Thompson. AGI, acute gastrointestinal illness. †Group 2: serotypes such as Bareilly, Gaminara, Give, Mississippi, Norwich, Pomona, Rubislaw, Tennessee, Urbana, Weltevreden. ‡Clinical manifestations of interest for initial elicitation were bacteremia and wound infections.

#### Transmission Pathway Definitions

We used definitions for 5 major pathways that were mutually exclusive and comprehensive (i.e., covering 100% of transmission modes) and that reflect those used by CDC for outbreak surveillance (*15**,**16*; Tables 2, 3). We defined 3 mutually exclusive waterborne subpathways (recreational water, drinking water, and nonrecreational nondrinking water) that were comprehensive (i.e., all waterborne pathway transmission fell into 1 of the 3 subpathways). We also defined and elicited 1 foodborne (food handler-related) and 2 environmental (presumed animal associated, presumed person-to-person) subpathways that accounted for only a portion of transmission within their main pathway. We calculated the unelicited proportion remaining of their respective main pathways during analysis and assigned it to the subpathways other foodborne and other environmental. For all transmission pathways, we defined the point of attribution as the point of exposure (i.e., the event during which a person ingested, or was otherwise exposed to, the pathogen).

**Table 2:** Major transmission pathway definitions, structured expert judgment, United States, 2017

Major transmission pathways	Description
Foodborne	Transmission occurs through eating food. Contamination can originate anywhere in the food production chain from primary production, to retail, and then to the home or restaurant. This pathway applies to all nonwater beverages and items ingested by humans as food (e.g., including raw milk and excluding items consumed for medicinal purposes).
Waterborne	Transmission occurs through the consumption of or direct contact with water or inhalation of aerosols originating from water. This includes drinking water, bottled water, recreational water (treated and untreated), and other water sources, such as water within buildings, used in medical devices, or for industry/manufacturing.
Person-to-person	Transmission occurs by direct contact with infected persons or their bodily fluids, or by contact with the local environment where an exposed person is simultaneously present with an infected person or visible excreta.
Animal contact	Transmission occurs through direct contact with an animal, its bodily fluids (excluding raw milk or other fluids consumed as food), fur, hair, feathers, scales, or skin, or by contact with the local environment where an infected animal, its visible excreta, fur, hair, feathers, scales, or skin was simultaneously present with the exposed person (e.g., barns, petting zoos, and pet stores). This pathway includes domestic animals, farm animals, wildlife, and pets.
Environmental	Transmission occurs through exposure to naturally occurring agents (e.g., free-living ameba or radon) or contact with contaminated air, mud, soil, or other outdoor or indoor surfaces or objects not attributable to foodborne, waterborne, person-to-person, or animal contact transmission, as defined for this project.

**Table 3:** Transmission subpathway definitions, structured expert judgment, United States, 2017

Subpathway	Description
Foodborne subpathway	
Food handler–related	When food processed or prepared for others is contaminated by an infected person.

Waterborne subpathways	
Recreational water, treated or untreated	Water that is used for recreational activities, such as in an aquatic facility or natural body of water. Can be treated or untreated. Treated water has undergone a systematic disinfection process (e.g., chlorination and filtration) with the goal of maintaining good microbiologic quality for recreation; untreated water has not undergone a disinfection or treatment process to maintain good microbiological quality for recreation (e.g., lakes, rivers, oceans, and reservoirs).
Drinking water	Water that is used primarily for drinking but including other domestic uses, such as washing or showering; can come from a public water system, a private well, or commercially bottled sources.
Nonrecreational, nondrinking water	Water that is used for purposes other than recreation or drinking (e.g., for agriculture, industry, medical treatment, backcountry streams or flood waters). Agricultural water includes water that is used to grow fresh produce and sustain livestock. Industrial water includes water used during manufacturing or in cooling equipment. Medical water includes any water used within medical devices or water used for washing surgical tools and equipment, and water used for hydrotherapy. This subcategory does not include transmission that can be accounted for by another major pathway, such as food or animals

Environmental subpathways	
Presumed animal contact associated	When a person becomes ill from exposure to soil, mud, or surfaces contaminated by an animal without direct contact or simultaneous presence with the animal, or when an infection is suspected to be animal associated because of previous knowledge about the pathogen.
Presumed person-to-person associated	When a person becomes ill from an exposure indirectly associated with an ill person.

#### Expert Identification and Selection

**Figure 1:**
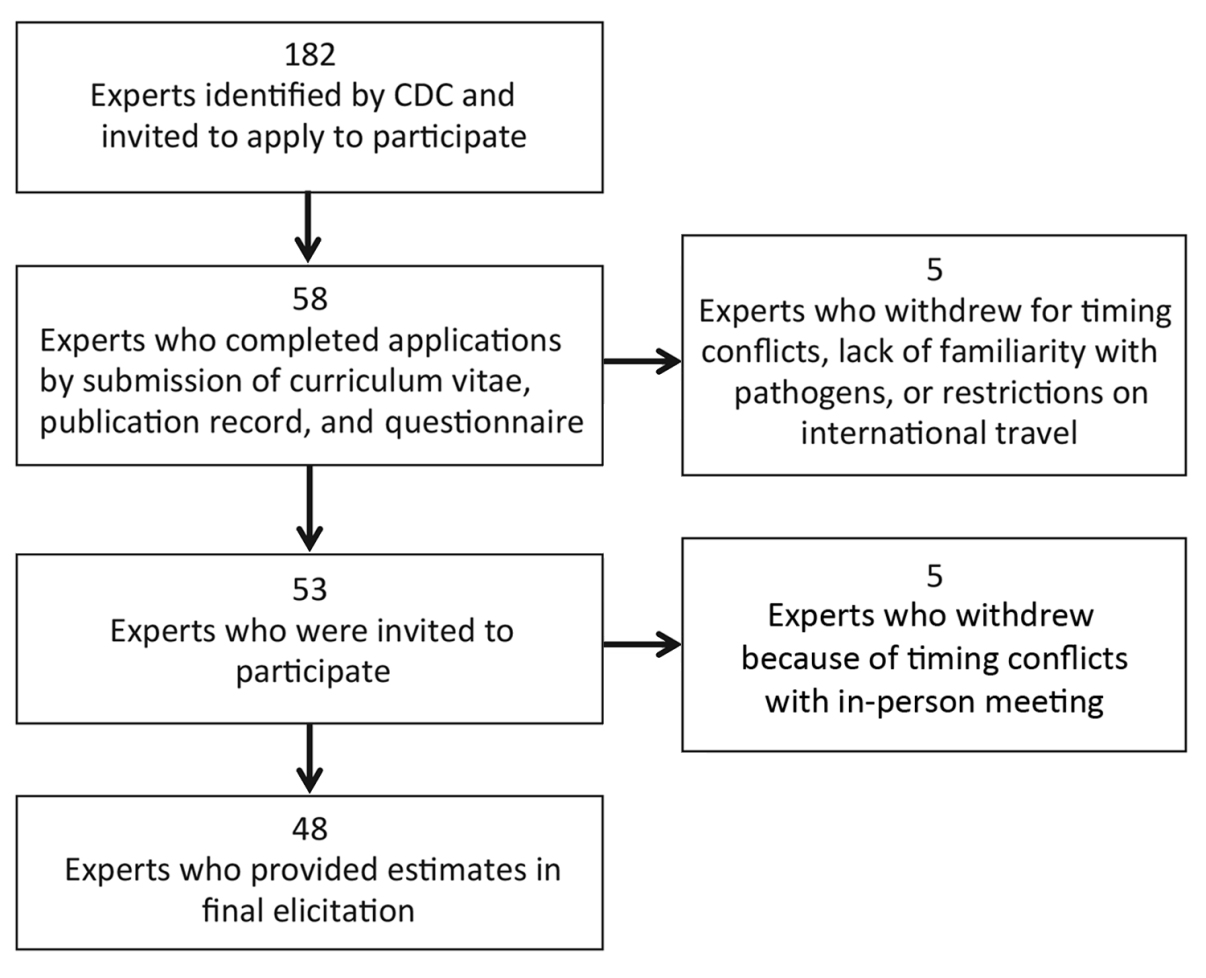
Expert selection process for study of attribution of illnesses transmitted by food and water to comprehensive transmission pathways using structured expert judgment, United States, 2017.

We identified 182 experts representing a range of scientific backgrounds (e.g., epidemiologists, laboratory scientists, and environmental engineers from government, academia, nongovernmental organizations, and industry) on the basis of publication records, experience, expertise, or previous participation in source attribution studies. We contacted the experts directly and invited them to apply for participation (Figure 1). Fifty-eight returned a curriculum vitae and publication record and completed a questionnaire about their professional interest, knowledge, and experience for each of the 33 pathogens using a 4-level Likert scale (high, medium, low, or none) by the requested deadline. We asked experts to suggest additional experts to be considered; the 3 who were suggested were also invited.

#### Assignment to Panels

We evaluated expert applications based on area of expertise, education, work history, professional interest, experience, and knowledge of the individual pathogens in this study. Publication record was not used to determine eligibility because it could have led to elimination of qualified experts who do not publish frequently. We used maximum bipartite matching in R version 3.3.1 with the igraph package version 1.0.1 to assign experts to panels based on their curricula vitae, publication records, and questionnaire responses (*17*,*18*). Final assignment ensured that experts were not on pathogen panels for which they reported none or low experience. Individual experts were on panels for <15 pathogens (Appendix 1).

#### Calibration Questions

The study administrators used unpublished data to develop calibration questions (Appendix 2). We developed 14 questions to evaluate the experts’ statistical accuracy and informativeness by probing the experts’ ability to provide reliable estimates under uncertainty. The subject domain of the questions aimed to represent expertise in public health surveillance of foodborne and waterborne diseases, food consumption patterns in the United States, and human exposure and occurrence data about pathogens in food, water, and the environment.

#### Target Questions

Target questions asked the proportion of illnesses transmitted through the 5 major pathways and 6 subpathways for all study pathogens. Study administrators blocked transmission pathways and subpathways for some pathogens based on their microbiology and ecology (Table 4). We created individualized Microsoft Excel version 14.7.7 (http://www.microsoft.com) files with separate sheets for calibration questions, target questions for each assigned pathogen, and additional instructions for each expert. We included verification aids in the worksheets to assist the experts (Appendix 3).

**Table 4:** Source attribution results for major transmission pathways, structured expert judgment, United States, 2017*

Pathogen name	Mean % (95% uncertainty interval)				
	Foodborne	Waterborne	Person-to-person	Animal contact	Environmental
Bacteria					
*Brucella* spp.	45 (13–77)	10 (0–42)	Blocked	36 (10–73)	9 (0–32)
*Campylobacter* spp.	57 (30–80)	13 (1–31)	7 (0–23)	16 (3–35)	7 (0–30)
Enterotoxigenic *Escherichia coli*	69 (37– 91)	9 (0–38)	7 (0–38)	Blocked	15 (2–33)
STEC O157	60 (40–77)	5 (1–13)	16 (4–33)	12 (3–25)	7 (1–17)
STEC non-O157	50 (26–75)	6 (0–17)	15 (2–34)	21 (2–46)	8 (0–24)
*E. coli*, other diarrheagenic	55 (27–80)	9 (0–30)	16 (2–39)	9 (0–33)	12 (0–33)
*Legionella* spp.	Blocked	97 (67–100)	0 (0–1)	Blocked	2 (0–28)
* Mycobacterium bovis*	75 (36–98)	1 (0–9)	9 (0–39)	13 (0–50)	2 (0–12)
Nontuberculous *Mycobacterium* spp.	Blocked	72 (39–94)	4 (0–21)	2 (0–35)	22 (0–49)
*Pseudomonas* spp., otitis externa	Blocked	81 (67–95)	3 (0–13)	1 (0–4)	15 (1–,25)
*Pseudomonas* spp., septicemia	Blocked	22 (3–53)	2 (0–19)	2 (0–11)	74 (41–94)
*Pseudomonas* spp., pneumonia	Blocked	51 (14–80)	4 (1–32)	0 (0–2)	45 (15–80)
*Salmonella enterica*, nontyphoidal	66 (48–81)	6 (0–22)	7 (0–16)	11 (3–24)	9 (2–21)
*S. enterica*, nontyphoidal, age <5 y	46 (20–66)	7 (0–26)	18 (6–35)	13 (2–30)	16 (2–36)
*S. enterica* serotype Enteritidis	80 (63–92)	4 (0–11)	7 (1–16)	5 (0–19)	4 (1–14)
*S. enterica* serotype I 4,[5],12:i:-	66 (40–82)	6 (1–15)	8 (1–17)	12 (2–27)	7 (0–20)
*S. enterica* serotype Javiana	56 (29–76)	7 (1–20)	9 (2–22)	14 (3–33)	14 (2–29)
*S. enterica* serotype Newport	74 (50–86)	2 (0–9)	7 (1–16)	8 (1–19)	8 (2–18)
*S. enterica* serotype Typhimurium	59 (27–78)	7 (1–18)	8 (2–19)	14 (3–29)	13 (2–30)
*S. enterica*, all other serotypes group 1	60 (29–79)	6 (1–18)	9 (2–21)	12 (2–29)	12 (3–,29)
*S. enterica*, all other serotypes group 2	40 (10–65)	7 (1–24)	10 (2–26)	17 (1–40)	26 (6–51)
*Shigella* spp.	8 (1–36)	4 (1–21)	81 (48–93)	Blocked	6 (0–26)
* Staphylococcus aureus*	Blocked	75 (23–98)	18 (1–71)	1 (0–5)	5 (0–37)
*Streptococcus* spp., group A	4 (0–33)	1 (0–6)	92 (55–99)	1 (0–12)	2 (0–19)
* Vibrio alginolyticus*	60 (24–84)	37 (13–71)	0 (0–1)	1 (0–4)	2 (0–11)
*V. alginolyticus*, non-AGI	2 (0–17)	97 (79–100)	0 (0–1)	0 (0–2)	0 (0–2)
*V. cholerae* nontoxigenic	92 (61–100)	6 (0–30)	1 (0–3)	0 (0–4)	0 (0–3)
*V. cholerae* nontoxigenic, non-AGI	33 (8–59)	65 (39–90)	0 (0–1)	0 (0–1)	2 (0–13)
* V. parahaemolyticus*	74 (59–91)	24 (7–38)	0 (0–2)	0 (0–2)	1 (0 –5)
*V. parahaemolyticus*, non-AGI	8 (2–39)	90 (57–97)	0 (0–1)	0 (0–1)	2 (0–8)
*V. vulnificus*†	20 (7–54)	77 (40–91)	0 (0–3)	1 (0–9)	2 (0–12)
*V. vulnificus*, non-AGI	20 (9–34)	78 (58–89)	0 (0–1)	1 (0–16)	2 (0–9)
*Vibrio* spp., other AGI	96 (69–100)	2 (0–23)	0 (0–1)	0 (0–2)	1 (0–8)
*Vibrio* spp, other non-AGI	95 (58–100)	3 (0–27)	0 (0–1)	0 (0–2)	2 (0–15)
* Yersinia enterocolitica*	77 (44–100)	9 (0–37)	3 (0–17)	4 (0–16)	8 (0–33)

Protozoa					
*Acanthamoeba* spp.	Blocked	82 (46–100)	Blocked	0 (0–0)	18 (0–54)
* Balamuthia mandrillaris*	Blocked	54 (5–95)	Blocked	0 (0–0)	46 (5–95)
*Cryptosporidium* spp.	7 (0–25)	43 (17–73)	20 (2–49)	21 (4–48)	8 (0–34)
* Cyclospora cayetanensis*	83 (59–99)	6 (0–25)	3 (0–14)	1 (0–9)	7 (0–28)
*Giardia* spp.	10 (0–35)	44 (16–78)	27 (3–59)	10 (0–38)	8 (0–37)
* Naegleria fowleri*	Blocked	88 (61–100)	Blocked	Blocked	12 (0–38)
* Toxoplasma gondii*	28 (4–60)	5 (0–27)	Blocked	58 (24–86)	9 (0–29)

Viruses					
Astrovirus	15 (1–38)	6 (0–25)	73 (44–94)	Blocked	6 (0–18)
Hepatitis A virus	42 (9–78)	8 (0–33)	41 (8–77)	Blocked	8 (0–34)
Norovirus	19 (6–37)	6 (0–25)	70 (46–88)	Blocked	5 (0–18)
Rotavirus	5 (0–20)	7 (0–28)	81 (57–98)	Blocked	5 (0–21)
Sapovirus	13 (0–34)	8 (0–30)	75 (49–94)	Blocked	4 (0–16)

*Blocked indicates pathways blocked by study administrators. AGI, acute gastrointestinal disease; STEC, Shiga toxin–producing *Escherichia coli*. †Clinical manifestations of interest for initial elicitation were bacteremia and wound infections.

#### Dry Run Exercise

We conducted a dry run exercise using video web conferencing to assess calibration questions, target question answer sheets, and expert training materials for completeness, clarity, and ease of use. Six persons from academia, state health departments, and CDC participated in this trial exercise, but not in the formal elicitation itself. We modified the elicitation materials based on feedback from this exercise.

#### Expert Orientation

Before the formal elicitation, experts attended a training webinar to learn definitions of transmission pathways, subpathways, and point of attribution. To ensure common understanding of the definitions, experts completed a 20-question review of knowledge after the webinar (Appendix 4).

We provided a background document summarizing current surveillance data, when available, and relevant research findings for each pathogen. The document contained links to selected research articles. Experts were encouraged to use any data they felt were informative to make their estimates; they were not limited to only this document.

### Elicitation

For the formal elicitation, 48 experts representing a wide range of professional and scientific backgrounds participated at a 2-day, in-person workshop in May 2017. During the workshop, experts participated in a 2-hour information session on probabilistic methods and providing estimates under uncertainty.

#### Calibration Questions

Experts were not expected to know true values precisely and provided low (5th percentile), median (50th percentile), and high (95th percentile) estimates to represent their uncertainty on the answers provided to the calibration questions. Experts were not allowed access to any additional resources while answering the calibration questions and, after they had they had finished, they could not return to this section to change their responses.

#### Target Questions

After completion of the calibration questions, experts provided 5th, 50th, and 95th percentile estimates for the proportion of domestically acquired illnesses that are transmitted through each major pathway and subpathway annually for each pathogen and target question in each panel to which they were assigned. The experts were also asked to indicate if they did not agree with the pathways blocked by study administrators. One pathway, person-to-person transmission for *Legionella* spp., was unblocked based on this feedback, and experts provided this estimate with the others at the in-person elicitation. Experts could access resources and discuss them with colleagues, if desired. However, we emphasized that the final estimates should represent the expert’s individual responses, not a group consensus.

### Postelicitation

#### Re-Elicitation

After the in-person elicitation was completed, we determined that re-elicitation for some pathogens was necessary. More granular detail was needed beyond the single estimate for *Pseudomonas*, so estimates were re-elicited for otitis externa, septicemia, and pneumonia. Based on feedback we received during the elicitation, we re-elicited estimates for non–acute gastrointestinal infections (non-AGI) for nontoxigenic *Vibrio cholerae*, *V. parahaemolyticus, V. vulnificus*, and *V. alginolyticus*. Experts were provided with feedback with updated surveillance data and given the opportunity to adjust their original estimates if new data led them to reconsider their previous estimates (Figure 1). The re-elicitations were completed through follow-up emails and web conferences.

#### Data Analysis

We analyzed data using EXCALIBUR (*19*). We combined all individual expert assessments by linear pooling into a single uncertainty assessment for each target question (*11*). For equal-based weighting, all experts’ assessments contributed to the combined uncertainty assessment evenly. We computed performance-based weighting by combining the statistical accuracy and information scores of experts in each panel. The weighted combination of experts is referred to as the decision maker. We used the item weight decision maker because this calculates and applies weights per individual target question rather than for all questions an expert answered. We performed optimization to determine the threshold by which an expert’s responses would be included in the final estimate or not. This was done separately per expert for each panel, based on each expert’s statistical accuracy score (*12*).

We performed a subgroup analysis to determine whether separate schools of thought existed as a result of experts’ self-identified background (categorized as mainly foodborne, mainly waterborne, or both). This analysis was completed by 2 independent reviewers who analyzed EXCALIBUR panel outputs for each target question to determine whether wide divergence existed among individual responses.

We normalized random samples from the weighted distributions for major transmission pathways and waterborne subpathways such that on each sample the values across pathways summed to 1. This process was done by resampling the cumulative distribution functions generated by EXCALIBUR 5,000 times in R version 3.4.3 for each pathogen, while dividing all sampled values by the sum of their values per iteration. Point estimates and 95% uncertainty intervals (UIs) for each target question and pathway were produced. We performed robustness analysis and out-of-sample validation to assess the performance of the method and to evaluate the effect of individual experts and individual calibration questions on the final distribution (Appendix 5) (*12*).

## Results

### Knowledge Review

The 20 questions were designed to be challenging, to emphasize application of the study definitions, and to represent scenarios at the boundaries among different transmission pathways. For 17 (85%) questions, >75% of participants answered with the correct major pathway, and of these questions, 13 (76%) were answered with the correct subpathway as well (Appendix 4).

### Major and Subpathway Results

**Figure 2:**
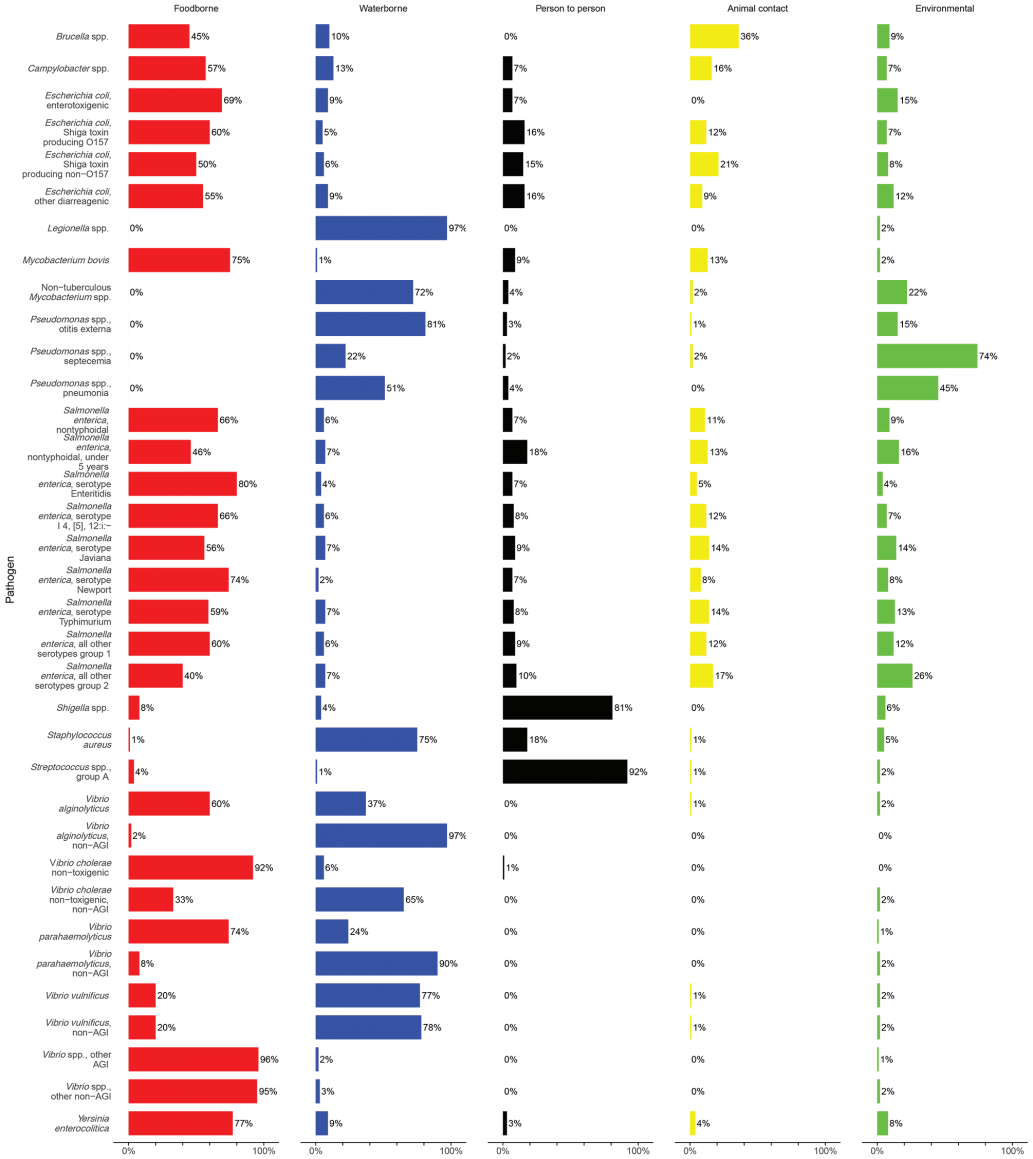
Source attribution results for major transmission pathways of bacteria in study of attribution of illnesses transmitted by food and water to comprehensive transmission pathways using structured expert judgment, United States, 2017.

**Figure 3:**
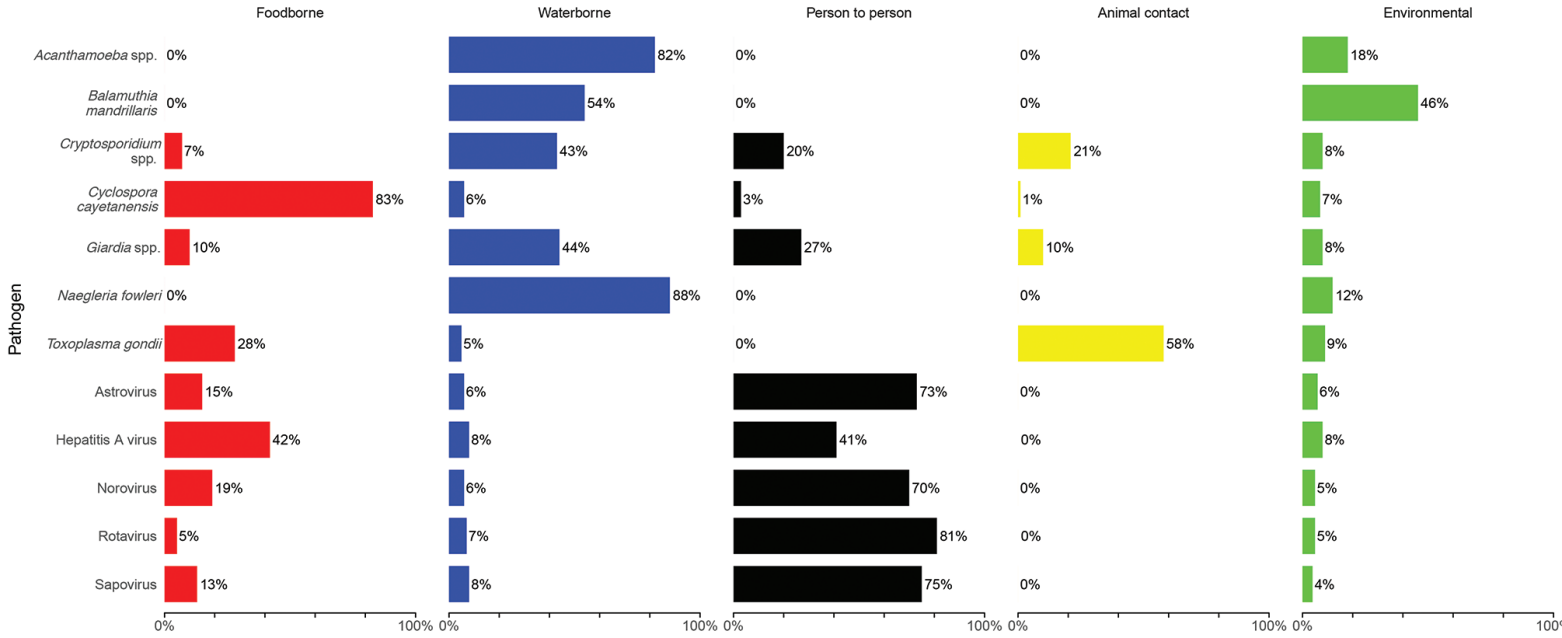
Source attribution results for major transmission pathways of protozoa and viruses for study of attribution of illnesses transmitted by food and water to comprehensive transmission pathways using structured expert judgment, United States, 2017.

Table 4 and Figures 2 and 3 show the proportion and UI of domestically acquired illnesses attributed to the 5 major transmission pathways; Tables 5 and 6 show the subpathway results. For all panels, a satisfactory number of accurate and informative experts were included. Differing schools of thought based on experts’ backgrounds were not identified (Appendix 5).

**Table 5:** Source attribution results for foodborne and environmental transmission subpathways, structured expert judgment, United States, 2017*

Pathogen name	Mean % (95% uncertainty interval)					
	Foodborne			Environmental		
	Food handler–related	Other foodborne		Presumed person-to-person	Presumed animal contact	Other environmental
Bacteria						
*Brucella* spp.	Blocked	100 (100–100)		Blocked	41 (2–96)	59 (4–98)
*Campylobacter* spp.	12 (0–58)	88 (42–100)		12 (0–46)	62 (3–100)	26 (0–89)
Enterotoxigenic *Escherichia coli*	23 (1–71)	77 (29–99)		8 (0–43)	Blocked	92 (54–100)
STEC O157	8 (0–55)	92 (45–100)		10 (0–46)	76 (16–100)	13 (0–73)
STEC non-O157	5 (0–29)	95 (71–100)		21(2–49)	65(19–91)	14 (0–55)
*E. coli*, other diarrheagenic	7 (0–54)	93 (46–100)		59 (3–100)	9 (0–39)	31 (0–91)
*Legionella* spp.	Blocked	Blocked		0 (0–6)	Blocked	99 (91–100)
* Mycobacterium bovis*	1 (0–13)	99 (87–100)		3 (0–34)	45 (0–100)	53 (0–100)
Nontuberculous *Mycobacterium* spp.	Blocked	Blocked		3 (0–35)	6 (0–87)	91 (0–100)
*Pseudomonas* spp., otitis externa	Blocked	Blocked		8 (0–51)	2 (0–11)	90 (16–100)
*Pseudomonas* spp., septicemia	Blocked	Blocked		9 (0–59)	1 (0–4)	91 (39–100)
*Pseudomonas* spp., pneumonia	Blocked	Blocked		10 (0–61)	1 (0–6)	88 (22–100)
*Salmonella enterica*, nontyphoidal	10 (0–38)	90 (62–100)		20(2–52)	45 (5–89)	35 (0–83)
*S. enterica*, nontyphoidal, under 5 y	10 (0–39)	90 (61–100)		35 (5–78)	45 (6–84)	20 (0–75)
*S. enterica* serotype Enteritidis	11 (0–51)	89 (49–100)		22 (2–56)	44 (3–88)	34 (0–84)
*S. enterica* serotype I 4,[5],12:i:-	10 (0–38)	90 (62–100)		21 (3–52)	45 (3–89)	34 (0–84)
*S. enterica* serotype Javiana	11 (0–48)	89 (52–100)		36 (4–80)	44 (5–84)	20 (0–75)
*S. enterica* serotype Newport	10 (0–39)	90 (61–100)		21 (3–53)	48 (5–89)	30 (0–82)
*S. enterica* serotype Typhimurium	10 (0–39)	90 (61–100)		21 (2–50)	49 (6–88)	31 (0–81)
*S. enterica*, all other serotypes group 1	10 (0–38)	90 (62–100)		21 (2–52)	48 (6–89)	31 (0–81)
*S. enterica*, all other serotypes group 2	10 (0–39)	90 (61–100)		35 (5–79)	44 (5–83)	20 (0–74)
*Shigella* spp.	71 (17–96)	29 (4–83)		90 (31–100)	Blocked	10 (0–69)
* Staphylococcus aureus*	Blocked	Blocked		76 (30–97)	3 (0–43)	21 (0–66)
*Streptococcus* spp., group A	51 (0–100)	49 (0–100)		94 (29–100)	2 (0–33)	4 (0–70)
*Vibrio alginolyticus,* AGI	5 (0–89)	95 (11–100)		2 (0–19)	2 (0–36)	96 (9–100)
*V. alginolyticus*, non-AGI	0 (0–2)	100 (98–100)		1 (0–3)	96 (45–100)	3 (0–54)
*V. cholerae* nontoxigenic AGI	1 (0–5)	99 (95–100)		6 (0–83)	9 (0–97)	85 (0–100)
*V. cholerae* nontoxigenic, non-AGI	0 (0–1)	100 (99–100)		1(0–4)	96 (26–100)	3(0–73)
*V. parahaemolyticus* AGI	5 (0–52)	95 (48–100)		2 (0–7)	2(0–24)	96 (18–100)
*V. parahaemolyticus*, non-AGI	0 (0–2)	100 (98–100)		1 (0–3)	96 (30–100)	3 (0–69)
*V. vulnificus*†	5 (0–72)	95 (28–100)		3 (0–48)	3 (0–50)	94 (0–100)
*V. vulnificus*, non-AGI	0 (0–2)	100 (98–100)		1 (0–3)	96 (29–100)	3 (0–70)
*Vibrio* spp., other AGI	3 (0–70)	97 (30–100)		1 (0–5)	2 (0–27)	96 (21–100)
*Vibrio* spp., other non-AGI	3 (0–43)	97 (57–100)		1 (0–2)	2 (0–31)	97 (38–100)
* Yersinia enterocolitica*	9 (0–55)	91 (45–100)		23 (0–67)	56 (8–99)	20 (0–82)
Protozoa						
*Acanthamoeba* spp.	Blocked	Blocked		Blocked	1 (0–6)	97 (45–100)
* Balamuthia mandrillaris*	Blocked	Blocked		Blocked	2 (0–12)	97 (37–100)
*Cryptosporidium* spp.	24 (0–87)	76 (13–100)		18 (0–61)	61 (7–99)	21(0–81)
* Cyclospora cayetanensis*	10 (0–68)	90 (32–100)		51 (0–100)	6 (0–70)	43 (0–100)
*Giardia* spp.	19 (0–72)	81 (28–100)		26 (1–66)	23 (0–86)	51 (0–97)
* Naegleria fowleri*	Blocked	Blocked		Blocked	Blocked	97 (47–100)
* Toxoplasma gondii*	Blocked	100 (100–100)		Blocked	80 (22–100)	20 (0–78)

Viruses						
Astrovirus	50 (0–100)	50 (0–100)		73 (1–100)	Blocked	27 (0–99)
Hepatitis A virus	48 (2–93)	52 (7–98)		86 (27–100)	Blocked	12 (0–72)
Norovirus	71 (29–99)	29 (1–71)		73 (2–100)	Blocked	27 (0–98)
Rotavirus	27 (0–98)	73 (2–100)		88 (35–100)	Blocked	11 (0–65)
Sapovirus	51 (0–99)	49 (1–100)		67 (0–100)	Blocked	33 (0–100)

*Blocked indicates pathways blocked by study administrators. AGI, acute gastrointestinal disease; STEC, Shiga toxin–producing *Escherichia coli*. †Clinical manifestations of interest for initial elicitation were bacteremia and wound infections.

**Table 6:** Source attribution results for waterborne transmission subpathways (means and 95 uncertainty interval), structured expert judgment, United States, 2017*

Pathogen name	Mean % (95% uncertainty interval)		
	Recreational water	Drinking water	Nonrecreational, nondrinking water
Bacteria			
*Brucella* spp.	45 (0–100)	8 (0–97)	47 (0–100)
*Campylobacter* spp.	32 (0–97)	44 (0–99)	24 (0–99)
Enterotoxigenic *Escherichia coli*	31 (3–85)	57 (8–94)	12 (0–58)
STEC O157	69 (33–94)	26 (3–60)	5 (0–28)
STEC non-O157	51 (18–77)	12 (0–43)	38 (12–69)
*E. coli*, other diarrheagenic	20 (2–53)	70 (34–92)	10 (0–38)
*Legionella* spp.	9 (2–35)	52 (19–78)	39 (13–69)
* Mycobacterium bovis*	21 (0–100)	14 (0–100)	65 (0–100)
Nontuberculous *Mycobacterium* spp.	13 (0–43)	67 (33–93)	20 (0–51)
*Pseudomonas* spp., otitis externa	95 (75–100)	3 (0–21)	2 (0–11)
*Pseudomonas* spp., septicemia	7 (2–37)	16 (1–50)	77 (37–94)
*Pseudomonas* spp., pneumonia	48 (17–74)	6 (1–33)	46 (18–76)
*Salmonella enterica*, nontyphoidal	18 (2–53)	75 (37–93)	7 (0–26)
*S. enterica*, nontyphoidal, <5 y	19 (3–49)	69 (38–91)	12 (1–30)
*S. enterica* serotype Enteritidis	20 (3–49)	71 (38–92)	9 (1–27)
*S. enterica* serotype I 4,[5],12:i:-	18 (2–49)	74 (38–93)	9 (0–35)
*S. enterica* serotype Javiana	21 (3–53)	67 (29–90)	12 (0–42)
*S. enterica* serotype Newport	17 (2–48)	74 (40–94)	9 (0–39)
*S. enterica* serotype Typhimurium	19 (3–51)	73 (39–93)	8 (1–29)
*S. enterica*, all other serotypes group 1	19 (3–51)	72 (36–93)	9 (0–39)
*S. enterica*, all other serotypes group 2	19 (2–50)	69 (36–91)	12 (1–40)
*Shigella* spp.	77 (41–95)	3 (0–25)	20 (3–50)
* Staphylococcus aureus*	91 (50–100)	5 (0–29)	4 (0–43)
*Streptococcus* spp., group A	73 (0–100)	10 (0–95)	18 (0–100)
*Vibrio alginolyticus* AGI	97 (66–100)	1 (0–6)	2 (0–21)
*V. alginolyticus*, non-AGI	96 (49–100)	2 (0–36)	3 (0–47)
*V. cholerae* nontoxigenic AGI	96 (56–100)	2 (0–11)	2 (0–22)
*V. cholerae* nontoxigenic, non-AGI	96 (50–100)	2 (0–14)	3 (0–43)
* V. parahaemolyticus*	98 (62–100)	1 (0–10)	1 (0–13)
*V. parahaemolyticus*, non-AGI	97 (50–100)	2 (0–35)	2 (0–37)
*V. vulnificus*†	98 (66–100)	1 (0–9)	2 (0–24)
*V. vulnificus*, non-AGI	96 (49–100)	2 (0–37)	2 (0–43)
*Vibrio* spp., other AGI	69 (0–100)	4 (0–69)	27 (0–100)
*Vibrio* spp, other non-AGI	70 (0–100)	4 (0–69)	26 (0–100)
* Yersinia enterocolitica*	51 (6–100)	28 (0–83)	21 (0–79)

Protozoa			
*Acanthamoeba* spp.	52 (8–88)	15 (0–51)	33 (3–76)
* Balamuthia mandrillaris*	48 (6–88)	4 (0–26)	48 (7–89)
*Cryptosporidium* spp.	66 (21–96)	24 (0–68)	11 (0–41)
* Cyclospora cayetanensis*	39 (0–99)	32 (0–97)	29 (0–100)
*Giardia* spp.	49 (9–93)	33 (2–82)	18 (0–67)
* Naegleria fowleri*	85 (51–98)	3 (0–27)	12 (1–45)
* Toxoplasma gondii*	37 (0–100)	27 (0–100)	36 (0–100)

Viruses			
Astrovirus	39 (0–99)	47 (0–100)	13 (0–92)
Hepatitis A virus	35 (0–100)	44 (0–100)	21 (0–97)
Norovirus	47 (8–90)	45 (6–86)	8 (0–42)
Rotavirus	41 (7–84)	50 (8–86)	9 (0–41)
Sapovirus	55 (11–97)	37 (0–84)	8 (0–41)

*AGI, acute gastrointestinal disease; STEC, Shiga toxin–producing *Escherichia coli*. †Clinical manifestations of interest for initial elicitation were bacteremia and wound infections.

#### Bacteria

Most of the pathogens in this study were bacteria; they encompassed 35 of the 47 target questions. More than half of transmission (>50%) was attributed to the foodborne pathway for *Campylobacter* spp.; enterotoxigenic *Escherichia coli*; Shiga toxin-producing *Escherichia coli* (STEC) O157; other diarrheagenic *E. coli*; *Mycobacterium bovis*; nontyphoidal *Salmonella enterica* (all ages and serotypes); *S. enterica* serotypes Enteritidis, I 4,[*5*],12:i:-, Javiana, Newport, Typhimurium, and group 1 serotypes; *Vibrio alginolyticus*; *V. cholerae* nontoxigenic; *V. parahaemolyticus; Vibrio* spp., other AGI; *Vibrio* spp, other non-AGI; and *Yersinia enterocolitica*. In addition, *Legionella* spp.; nontuberculous *Mycobacterium* spp.; *Pseudomonas* spp., otitis externa; invasive *Staphylococcus aureus*; *V. alginolyticus*, non-AGI; *V. cholerae* nontoxigenic, non-AGI; *V. parahaemolyticus*, non-AGI; and *V. vulnificus* were all estimated to have majority transmission from the waterborne pathway. Most transmission for *Shigella* spp. and group A *Streptococcus* were estimated to be through person-to-person transmission. No bacterial pathogen had majority transmission through animal contact. *Pseudomonas* spp. septicemia was attributed primarily to the environmental pathway.

#### Protozoa

*Cyclospora cayetanensis* was the only protozoan estimated to have majority transmission through the foodborne pathway. *Acanthamoeba* spp. and *Naegleria fowleri* both had >80% transmission attributed to the waterborne pathway, and 54% (UI 5%–95%) of *Balamuthia mandrillaris* infections were estimated to occur through waterborne transmission. No protozoa had majority person-to-person or environmental transmission. Waterborne transmission was estimated at 43% (UI 17%–73%) for *Cryptosporidium* spp. and 44% (UI 16%–78%) for *Giardia* spp. Among all pathogens, *Toxoplasma gondii* had the highest attribution to animal contact transmission, 58% (UI 24%–86%).

#### Viruses

Most transmission for astrovirus, norovirus, rotavirus, and sapovirus was attributed to the person-to-person pathway. Hepatitis A virus was estimated to have the highest proportion of illness transmitted by the foodborne pathway at 42% (UI 9%–78%). Of this, 48% (UI 2%–93%) was considered food handler related. Of foodborne transmission, 50%–71% was estimated to be food handler related for astrovirus, norovirus, and sapovirus. For all viruses, 67%–88% of environmental transmission was attributed to the subpathway of presumed person-to-person transmission.

## Discussion

This study presents a novel method for estimating the proportion of illnesses from pathogens transmitted commonly by food and water in the United States through comprehensive and mutually exclusive pathways. It includes estimates for food handler–related, recreational water, drinking water, nonrecreational nondrinking water, and various environmental subpathways. This method enabled estimates to be informed by multiple data sources, including outbreak surveillance data, studies of sporadic illnesses, case reports, and experts’ professional knowledge. The use of calibration to weight expert responses is a distinguishing characteristic of the classical model and introduces mathematical rigor not found with other elicitation methods.

Similar SEJ studies have been conducted in numerous countries, including Australia, Canada, and the Netherlands, as well as for global subregions, by the World Health Organization. Each of these used different transmission pathway definitions, study designs, and elicitation methods (*20*–*23*). This and other variations in methods limit comparison of estimates across studies, but provide support for some of the differences between our study results and previous US pathway attribution estimates. Previous estimates of foodborne transmission for 33 pathogens and animal contact transmission for 6 pathogens included in our study are available (*2*,*24*). We compared published foodborne and waterborne attribution studies with this study (Tables 7, 8).

**Table 7:** Comparison of proportion of illnesses attributed to foodborne transmission from this and earlier studies*

Details	Study					
	Scallan et al. (*2*)	Hald et al. (*20*)	Havelaar et al. (*21*)	Butler et al. (*22*)	Vally et al. (*23*)	This study
Country	United States	AMR A (Canada, Cuba, USA)	Netherlands	Canada	Australia	United States
Type	Outbreak surveillance data or published studies	SEJ	SEJ	SEJ	SEJ	SEJ

Bacteria						
*Brucella* spp.	50	75	NE	34.6	NE	45
*Campylobacter* spp.	80	73	42	62.3	76	57
STEC O157	68	59	40	61.4	Combined as all STEC, 55	60
STEC non-O157	82	NE	42	59.7	Combined as all STEC, 55	50
Enterotoxigenic *Escherichia coli*	100 (only foodborne)	36	NE	44.4	Combined as other pathogenic *E. coli*, 24	69
*E. coli*, other diarrheagenic	30	NE	NE	41	Combined as other pathogenic *E. coli*, 24	55
* Mycobacterium bovis*	95	NE	NE	NE	NE	75
*Salmonella* spp.	94	73	55	62.9	71	66
*Shigella* spp.	31	12	NE	25.9	11	8
* Vibrio vulnificus*	47	NE	NE	70.6	NE	Non-AGI, 20
* Vibrio parahaemolyticus*	86	NE	NE	82.8	NE	AGI, 74 Non-AGI, 8
*Vibrio* spp. other	57	NE	NE	88.9	NE	AGI, 96 Non-AGI-95
* Yersinia enterocolitica*	90	NE	NE	82.8	NE	77
Protozoa						
*Cryptosporidium* spp.	8	16	12	11.3	NE	7
* Cyclospora cayetanensis*	99	NE	NE	83.1	NE	83
*Giardia* spp.	7	11	13	7.2	NE	10
* Toxoplasma gondii*	50	60	56	51.4	NE	28

Viruses						
Astrovirus	<1	NE	NE	9.9	NE	15
Hepatitis A virus	7	42	11	29.5	12	42
Norovirus	26	23	17	18.4	17	19
Rotavirus	<1	NE	13	7.3	NE	5
Sapovirus	<1	NE	NE	16.9	NE	13

*NE, not estimated; SEJ, structured expert judgment; STEC, Shiga toxin–producing *Escherichia coli*.

**Table 8:** Comparison of proportion of illnesses attributed to waterborne transmission from this and earlier published studies*

Details	Study			
	Hald et al. (*20*)	Butler et al. (*22*)	Vally et al. (*23*)	This study
Country	AMR A (Canada, Cuba, USA)	Canada	Australia	United States
Type	SEJ	SEJ	SEJ	SEJ

Bacteria				
*Brucella* spp.	1	4	NE	10
*Campylobacter* spp.	11	9.3	6	13
STEC O157	7	13.3	Combined as all STEC, 8	5
STEC non-O157	NE	11.4	Combined as all STEC, 8	6
Enterotoxigenic *Escherichia coli*	42	15.3	Combined as other *E. coli*, 14	9
*E. coli*, other diarrheagenic	NE	15.6	Combined as other *E. coli*, 14	9
*Salmonella* spp.	2	8	5	6
*Shigella* spp.	10	12.2	4	4
* Vibrio vulnificus*	NE	23.2	NE	Non-AGI, 78
* V. parahaemolyticus*	NE	11	NE	AGI, 24; non-AGI, 90
*Vibrio* spp. other	NE	7.6	NE	AGI, 2; non-AGI, 3

Protozoa				
*Cryptosporidium* spp.	37	36.8	NE	43
* Cyclospora cayetanensis*	NE	7.7	NE	6
*Giardia* spp.	42	NE	NE	44
* Toxoplasma gondii*	19	8.8	NE	5

Viruses				
Astrovirus	NE	6.8	NE	6
Hepatitis A virus	1	6.2	4	8
Norovirus	22	7.4	3	6
Rotavirus	NE	5.9	NE	7
Sapovirus	NE	1.4	NE	8

*NE, not estimated; SEJ, structured expert judgment; STEC, Shiga toxin–producing *Escherichia coli*.

Differences from previously published work on foodborne transmission attribution proportions were noted, including for *Campylobacter* spp., STEC non-O157, other diarrheagenic *E. coli*, nontyphoidal *S. enterica*, *M. bovis, Shigella* spp., *Y. enterocolitica, C. cayetanensis, T. gondii,* astrovirus, rotavirus, sapovirus, and hepatitis A virus. These differences could be the result of changes in data availability or analytic methods. For example, previous US foodborne illness estimates used data from surveillance, risk factor studies, and literature review (*2*). Based on available data for *S. enterica* (a case-control study of sporadic illness and unpublished outbreak data [*2**,**25*]), a study used an estimate of 94% foodborne transmission, notably higher than this study’s estimate of 66% (UI 48%–81%). Estimates more similar to the current study were reported in SEJ studies in the Netherlands (55%), Canada (63%), and Australia (71%) (*21*,*22*); these studies examined attribution to similar major pathways to those included in this study versus foodborne transmission only. Our estimates of foodborne transmission of astrovirus (15%), rotavirus (5%), and sapovirus (13%) are much higher than the estimate of <1% for each in an earlier study (*2*); reports of foodborne outbreaks caused by these viruses in CDC’s outbreak surveillance systems informed our estimates. Reporting of enteric disease outbreaks transmitted by nonfoodborne routes has improved, and experts probably used these new data to inform their estimates (*26*).

This study provides noteworthy estimates for the food handler–related subpathway. For hepatitis A, both the World Health Organization and this study estimate 42% foodborne transmission, of which this study estimated 48% (UI 2%–93%) to be food handler-related (*20*). However, this study was conducted before widespread awareness of a massive increase in person-to-person transmission in the United States (*27*). Previous estimates of foodborne transmission were 11% in the Netherlands and 7% in the United States (*2*,*21*). The use of different pathway definitions, points of attribution, and inclusion of travel-related illness in these other studies might have contributed to these differences (*21*,*28*). For norovirus, 71% (UI 29%–99%) of foodborne transmission in our study was attributed to the food handler subpathway, which is supported by studies of outbreaks in the United States (*29*,*30*).

For the waterborne transmission pathway, attribution in the context of the other pathways has not been done before in the United States. Furthermore, these estimates include subpathway estimates and non-gastroenteritis clinical outcomes. For bacterial pathogens, the estimates suggest that the proportion of illnesses linked to water is higher than previously appreciated. The estimates for waterborne bacterial pathogens were associated with high rates of illness and death, including nontuberculous *Mycobacterium* spp., *Pseudomonas* spp., and *Legionella* spp. Of note, neither *Giardia* spp. nor *Cryptosporidium* spp., parasites traditionally understood to be waterborne, were assessed as predominantly waterborne; instead, person-to-person and animal contact, particularly for *Cryptosporidium*, were key pathways. For the free-living amebae *Acanthamoeba* spp., *B. mandrillaris*, and *N. fowleri*, limited data are available on exact exposures associated with these rare illnesses (*31*,*32*). The proportion of viral pathogens transmitted by water was estimated to be relatively low (6%–8%), although for norovirus this represents a substantial proportion of estimated annual waterborne disease illnesses (*32*). This study also provides estimates for 3 waterborne disease subpathways. Of note is the proportion of otitis externa infections caused by *Pseudomonas* spp. that were attributed to recreational water exposure, and the combined contribution of drinking and nonrecreational, nondrinking water exposures to nongastroenteritis outcomes of *Pseudomonas* spp. (excluding otitis externa), nontuberculous *Mycobacterium* spp., and *Legionella* spp. CDC has used results from this SEJ to help estimate that 7.2 million waterborne illnesses occur from 17 pathogens annually, including 600,000 emergency department visits, 120,000 hospitalizations, and 7,000 deaths, incurring $3.2 billion (2014 US dollars) in direct healthcare costs (*33*).

Whereas the primary focus of this SEJ study was illnesses transmitted commonly by food and water, including person-to-person, animal contact, and environmental transmission was integral to the study and led to notable findings. For example, this study estimated animal contact transmission of STEC O157 at 12% (UI 3%–25%) and of STEC non-O157 at 21% (UI 2%–46%). Previous US animal contact estimates, which were based on a FoodNet case-control study and outbreak surveillance data, estimated STEC O157 at 6% and STEC non-O157 at 8% (*24*). This discrepancy may be the result of differences in pathway definitions and the inclusion of additional data.

As with other SEJ studies, this study is subject to limitations that can affect the interpretation of results. Estimates for many pathogens had wide UIs, highlighting areas in which data gaps remain and further investment into public health surveillance and research may be warranted. More detailed attribution, such as by food category, was beyond the scope of this study. This study considered attribution at a national level and does not represent the geographic variability that exists for some pathogens. Experts provided estimates considering data available during the elicitation session, but infectious disease epidemiology can change rapidly, so these results may not reflect current transmission patterns. New information should be considered when applying these estimates (e.g., for disease burden calculations). Expert fatigue may have been a factor for participants who were asked to provide estimates for a large number of pathogens. For intervention and policy-making purposes, these results should be considered in context with results from other data-driven approaches, such as those done by the Interagency Food Safety Analytics Collaboration and for the Model Aquatic Health Code (*34*,*35*).

In conclusion, our findings provide a balanced understanding of multiple routes of transmission for 33 pathogens. This information can be used to support appropriate targeting of resources to prevent infections transmitted by all pathways and to invest in research and surveillance.

Appendix 1 Additional information about assigning pathogens to experts for structured expert judgment for attribution of foodborne and waterborne illnesses to comprehensive transmission pathways.

Appendix 2 Additional information about calibration questions for structured expert judgment for attribution of foodborne and waterborne illnesses to comprehensive transmission pathways.

Appendix 3 Additional tables about the various pathogens for structured expert judgment for attribution of foodborne and waterborne illnesses to comprehensive transmission pathways.

Appendix 4 Additional information about the knowledge review questionnaire and its results for structured expert judgment for attribution of foodborne and waterborne illnesses to comprehensive transmission pathways.

Appendix 5 Additional discussion about the validation analysis for structured expert judgment for attribution of foodborne and waterborne illnesses to comprehensive transmission pathways.
