# Supplementary material for: Attribution of Illnesses Transmitted by Food and Water to Comprehensive Transmission Pathways Using Structured Expert Judgment, United States
Source: Emerg Infect Dis. 2021 Jan;27(1):182–95. doi: 10.3201/eid2701.200316 (PMC7774530; doi:10.3201/eid2701.200316)
Supplement: Appendix 1 — Additional information about assigning pathogens to experts for structured expert judgment for attribution of foodborne and waterborne illnesses to comprehensive transmission pathways. [file 20-0316-Techapp-s1.pdf]

Article DOI: https://doi.org/10.3201/eid2701.200316

Attribution of Illnesses Transmitted by Food and Water to Comprehensive Transmission Pathways Using Structured Expert Judgment, United States

Appendix 1

Assigning Pathogens to Experts

Experts were polled with the question “Please indicate your professional interest, knowledge, and experience for each pathogen” for each of the 33 pathogens of interest. Answers were given on a Likert scale of high, medium, low, or none. Because we asked about professional interest, knowledge, and experience, as opposed to asking for self-ranked expertise, experts were able to indicate pathogens for which they would feel most able to provide estimates.

To support the assignment, we grouped pathogens into 15 panels with similar characteristics regarding microbiology, ecology, and/or transmission patterns, as follows:

- *Acanthamoeba* spp., *Balamuthia mandriallis*, *Naegleria fowleri*
- Astrovirus, norovirus, rotavirus, sapovirus
- *Brucella* spp., *Mycobacterium bovis*
- *Campylobacter* spp., *Yersinia enterocolitica*
- *Cryptosporidium* spp., *Giardia* spp.
- *Cyclospora cayetenensis*
- Enterotoxigenic *Escherichia coli*, other diarrheagenic *Escherichia coli*, *Shigella* spp.
- Hepatitis A virus
- *Legionella*, nontuberculous *Mycobacterium bovis*
- *Pseudomonas* spp.

- *Salmonella enterica*, nontyphoidal (estimates will be requested for all serotypes, as well as separately for serotypes Enteritidis, Typhimurium, Newport, i4, [5], 12:i:-, Javiana and other serotypes groups 1 and 2)
- Shiga toxin-producing *Escherichia coli* non-O157, Shiga toxin-producing *Escherichia coli* O157
- *Staphylococcus aureus* (invasive), *Streptococcus* spp., group A
- *Toxoplasma gondii*
- *Vibrio cholerae* (nontoxogenic), *Vibrio parahaemolyticus*, *Vibrio vulnificus*, *Vibrio* spp., other

Self-ratings were converted to numeric scores (0 = none, 1 = low, 2 = medium, 3 = high). The four-point Likert scale was not sufficiently informative for the algorithm used; additional information to support the assignment was based on indications of special expertise for particular pathogens. Two points were added to the expert's self-rating for any pathogen(s) about which he or she had distinctive expertise based on review of his or her curriculum vitae or publication record by the elicitation team. An average score by expert and major pathogen group (i.e., bacteria, viruses, protozoa) was calculated, and half of the average score was added to each related pathogen specific score to promote greater grouping by major pathogen group for experts. Average scores were then calculated based on the 15 sets listed previously.

Using these scores, we assigned experts to pathogens in rounds by determining the maximum bipartite graph (node type 1: expert; node type 2: pathogen set; edge weight: average set score) (*1*,*2*). This ensured that on each round the highest total score pairing of experts to pathogens was obtained. The edge order was randomly selected for each round to avoid potential issues with ties. The rounds proceeded until all matches were exhausted. The final panels were assigned based on filtering the results to include only experts with an average score of $\geq$1.5 for the pathogen set and limiting each expert to $\leq$15 pathogens.

References

1. Sedgewick R, Wayne K. Algorithms, 4th ed. Upper Saddle River (NJ): Addison-Wesley; 2011.

2. R Core Team. R: a language and environment for statistical computing. Vienna: R Foundation for Statistical Computing; 2017.
