# Supplementary material for: Attribution of Illnesses Transmitted by Food and Water to Comprehensive Transmission Pathways Using Structured Expert Judgment, United States
Source: Emerg Infect Dis. 2021 Jan;27(1):182–95. doi: 10.3201/eid2701.200316 (PMC7774530; doi:10.3201/eid2701.200316)
Supplement: Appendix 2 — Additional information about calibration questions for structured expert judgment for attribution of foodborne and waterborne illnesses to comprehensive transmission pathways. [file 20-0316-Techapp-s2.pdf]

Article DOI: https://doi.org/10.3201/eid2701.200316

# Attribution of Illnesses Transmitted by Food and Water to Comprehensive Transmission Pathways Using Structured Expert Judgment, United States

## Appendix 2

### Calibration Questions

### General Approach

Answers to calibration questions were required from the experts to provide weighting of their responses to the target variables. This procedure did not test experts' factual knowledge on the calibration questions, but rather their ability to provide valid estimates under uncertainty, specifically within the subject matter domain. This was done by asking for the experts' judgments of the low (5th percentile), median/best (50th percentile), and high (95th percentile) estimates that could be taken to represent their uncertainty distributions over the actual data values. The experts were not expected to know precisely these true values (but the study administrators did). However, they were expected to encompass the true values by providing suitable 90% uncertainty intervals and locate central tendency by an indicative median value. The median value need not be symmetric within the 90% uncertainty interval, but can indicate the expert's judgment of skewness (e.g., he or she might give 3 quantile values: [1; 5; 15] if he or she thought the uncertainty was right-skewed to higher values).

The initial strategy in the creation of the calibration questions was to include multiple domains in the calibration questions. The aim was to include questions that were relevant to the areas of expertise identified as desirable by CDC. The domains were as follows:

- Public health surveillance
- Occurrence data of food, water, and environmental hazards

- Exposure and frequency of exposure to hazards
- Food consumption patterns in the United States

The following expertise areas of interest were included on the expert questionnaire:

- Microbiology
- Bacteriology
- Virology
- Parasitology
- Enteric pathogens
- Epidemiology
- Public health
- Food safety
- Veterinary science
- Environmental microbiology

## Calibration Questions

Preceding each calibration question, a short description was provided to orient the experts to the data sources from which the questions were derived. The wording given here is as it was provided to the experts at the time of elicitation.

### FoodNet

The US Foodborne Diseases Active Surveillance Network, or FoodNet, has been tracking trends for infections commonly transmitted through food. This is done through active surveillance is in the following 10 US states: Connecticut, Georgia, Maryland, Minnesota, New Mexico, Oregon, Tennessee, and parts of California, Colorado, and New York. CDC releases preliminary data from the previous year annually, usually in the spring. The most recently available data were for 2015. Data for 2016 are expected to be published in April 2017.

Based on active surveillance data from FoodNet, what was the incidence (per 100,000 population) of laboratory-confirmed human *Cyclospora cayetanensis* infections for the year 2016?

Background information: In the year 2015, a total of 65 cases of *Cyclospora cayetanensis* were reported in the FoodNet database. This represents an incidence of 0.13 per 100,000 population.

| ____________ | ____________ | ____________ |
|---|---|---|
| Low (5th) | Median (50th) | High (95th) |

Based on active surveillance data from FoodNet, what was the incidence (per 100,000 population) of laboratory-confirmed human *Salmonella* infections for the year 2016?

Background information: In the year 2015, a total of 7,719 cases of human *Salmonella* were reported in the FoodNet database. This represents an incidence of 15.74 per 100,000 population.

| ____________ | ____________ | ____________ |
|---|---|---|
| Low (5th) | Median (50th) | High (95th) |

**NNDSS**

The US National Notifiable Disease Surveillance System (NNDSS) tracks all notifiable diseases that are reported to CDC by state and territorial jurisdictions. Each state has mandatory reporting criteria, but no federal-level reporting criteria exist. The state and territorial agencies voluntarily submit information to NNDSS, which the CDC oversees. The general system has been in existence since 1878. Notifiable disease surveillance is “passive” at the national level and is susceptible to underreporting. Annual reports are issued in July. The most recently available data were for 2015 and were published in *MMWR* in July 2016.

The annual number of human cases of acute hepatitis A reported to CDC through the NNDSS passive surveillance system has declined markedly over the past decade.
What was the percent decrease from 2013 to 2014 in the annual number of cases of hepatitis A reported to CDC through the NNDSS system?

This would be calculated as follows:
(number of cases in 2013 – number of cases in 2014)/(number of cases in 2013) × 100%

| | | |
|---|---|---|
| ___________ | ___________ | ___________ |
| Low (5th) | Median (50th) | High (95th) |

**NCOD**

The Environmental Protection Agency (EPA) is required to assemble and maintain national drinking water contaminant occurrence for 30 regulated and unregulated contaminants in public water systems. EPA tracks these data in the National Contaminant Occurrence Database (NCOD). This database was established in 1996 in accordance with the amendments to the Safe Drinking Water Act (SDWA). EPA maintains 2 data management systems for water quality information, the Legacy Data Center and STORET. These contain raw biologic, chemical, and physical data on surface and ground water collected by federal, state, and local agencies, academics, volunteer groups, tribes, and others. These reports are released in 3-year increments and published the following summer. The most recent data are from the years 2008–2015 and were published in July 2016.

EPA uses the Unregulated Contaminant Monitoring Rule (UCMR) program to collect data for certain contaminants. This monitoring covers a representative sample of public water systems (PWS) that serve $\leq$10,000 people in the United States. *E. coli* has a minimum reporting level (MRL) of 1 MPN[3]/100 mL according to NCOD. Between 2013 and 2015, a total of 1,045 samples were taken from these public water systems and tested for *E. coli*.

Based on the surveillance data in the NCOD, how many of these samples contained results with greater than or equal to the MRL for *E. coli* in 2016?

| | | |
|---|---|---|
| ___________ | ___________ | ___________ |
| Low (5th) | Median (50th) | High (95th) |

**NHANES**

The United States Department of Agriculture (USDA) publishes food consumption estimates of the average daily intake of food, by food source and demographic characteristics. These data were last updated in 2014 and include estimates from 2007–2010. These estimates are produced through the collection of data as part of the National Health and Nutrition Examination

Survey (NHANES). Data collection for these estimates began in 2003 and requires persons to record 2 nonconsecutive days using 24-hour dietary recall to obtain information about what they eat. Data on where food was purchased and eaten are included. NHANES data are released on a biannual basis for public use. NHANES oversamples from the underrepresented populations of African Americans, Hispanics, and persons $\geq$60 years of age.

NHANES includes fresh, canned, and frozen vegetables in its analysis of "total vegetables." This estimate does not include legumes.

Based on data collected by USDA for NHANES, what is the mean daily intake of total vegetables, in cups, for an individual, when considering the total US population age 2 and over for the year 2012?

| ____________ | ____________ | ____________ |
|---|---|---|
| Low (5th) | Median (50th) | High (95th) |

NHANES defines dairy products as fluid milk, cheese, and yogurt. Based on data collected by the USDA for NHANES, what is the mean daily intake of dairy, in cups, for a child in the US during 2012?

| ____________ | ____________ | ____________ |
|---|---|---|
| Low (5th) | Median (50th) | High (95th) |

**FSIS**

The Food Safety and Inspection Service (FSIS), as part of the United States Department of Agriculture (USDA), publishes data on the prevalence, volume weighted percent positive, or percent positive calculations for microbial pathogens in FSIS-regulated products. These results are released quarterly. These products include raw beef, raw pork, poultry, and ready-to-eat products. Pathogens tested for are *Salmonella*, *Campylobacter*, Shiga toxin-producing *E. coli* (STEC), *Listeria monocytogenes*, and chemical residues.

Between January 1, 2016 and December 31, 2016, a total of 11,277 samples of raw ground beef from 1,193 establishments were tested for *Salmonella* spp. Of these samples, how many tested positive for *Salmonella* spp.?

| | | |
|---|---|---|
| ____________ Low (5th) | ____________ Median (50th) | ____________ High (95th) |

**NARMS Background**

The National Antimicrobial Resistance Monitoring System (NARMS) is a national public health surveillance system that tracks changes in the antimicrobial susceptibility of certain enteric bacteria found in ill persons, retail meats, and food animals in the United States. NARMS was established in 1996 and is a collaboration among CDC, USDA, and FDA. Reports are published annually, representing data from 2 years prior. Thus, the report of data from 2014 was published in 2016.

NARMS tests *Salmonella* samples for resistance to 9 antimicrobial classes. These include aminoglycosides, β-lactam/β-lactamase inhibitor combinations, cephems, folate pathway inhibitors, macrolides, penicillins, phenicols, quinolones, and tetracyclines.

In 2014, a total of 2,127 *Salmonella* isolates from humans were tested by NARMS for resistance to the above antimicrobial agents.

What percentage of these samples showed no resistance to any of the antimicrobial agents tested?

| | | |
|---|---|---|
| ____________ Low (5th) | ____________ Median (50th) | ____________ High (95th) |

In 2014, a total of 4,122 Campylobacter isolates were tested in NARMS. Of these, 1,397 samples were from humans. What percentage of human samples tested in 2014 showed resistance to ciprofloxacin?

| | | |
|---|---|---|
| ____________ Low (5th) | ____________ Median (50th) | ____________ High (95th) |

**NORS**

The National Outbreak Reporting System (NORS) is a web-based platform used by local, state, and territorial health departments in the United States. This system is used to report all waterborne disease outbreaks, foodborne disease outbreaks, and enteric disease outbreaks transmitted by contact with environmental sources, infected persons or animals, or unknown modes of transmission. Data are evaluated continuously as outbreaks are reported into the system. Final data are typically released 12–18 months after the end of the reporting year.

As reported in 2014, between 2009 and 2010 there were 11 outbreaks involving harmful algal blooms (HABs). What percentage of individuals affected by the HAB outbreaks were hospitalized?

| ____________ | ____________ | ____________ |
|---|---|---|
| Low (5th) | Median (50th) | High (95th) |

A total of 864 foodborne disease outbreaks were reported in NORS for the year 2014. This includes both confirmed and suspected etiologies, as is reported annually. Of the outbreaks attributed to a single food category, how many were associated with chicken products?

| ____________ | ____________ | ____________ |
|---|---|---|
| Low (5th) | Median (50th) | High (95th) |

During 2014, there were 712 hospitalizations due to illnesses associated with NORS-reported outbreaks. How many hospitalizations due to illnesses associated with NORS-reported outbreaks were there in 2016?

| ____________ | ____________ | ____________ |
|---|---|---|
| Low (5th) | Median (50th) | High (95th) |

**Recreational Water Outbreaks Background**

The CDC defines recreational water as treated venues (e.g., pools, hot tubs, or spas) and untreated water venues (e.g., lakes and oceans). The Waterborne Disease and Outbreak Surveillance System collects data on waterborne diseases and outbreaks associated with recreational water, drinking water, environmental, and undetermined water exposures. Outbreaks in recreational water are reported in *MMWR* annually, reflecting finalized data from 3 years prior. Thus, the 2015 report reflects 2011–2012 data.

For the years 2009–2010, there were a total of 81 outbreaks attributed to recreational water (both treated and untreated) reported to the Waterborne Disease and Outbreak Surveillance System.

For the years 2011–2012, how many outbreaks were be attributed to untreated recreational water?

| ____________ | ____________ | ____________ |
| --- | --- | --- |
| Low ($5^{th}$) | Median ($50^{th}$) | High ($95^{th}$) |

For the years 2009–2010, there were a total of 81 outbreaks attributed to recreational water (both treated and untreated) reported to the Waterborne Disease and Outbreak Surveillance System.

What percentage of recreational water outbreaks for the years 2011–2012 were caused by *Cryptosporidium* species?

| ____________ | ____________ | ____________ |
| --- | --- | --- |
| Low ($5^{th}$) | Median ($50^{th}$) | High ($95^{th}$) |
