# Supplementary material for: Attribution of Illnesses Transmitted by Food and Water to Comprehensive Transmission Pathways Using Structured Expert Judgment, United States
Source: Emerg Infect Dis. 2021 Jan;27(1):182–95. doi: 10.3201/eid2701.200316 (PMC7774530; doi:10.3201/eid2701.200316)
Supplement: Appendix 4 — Additional information about the knowledge review questionnaire and its results for structured expert judgment for attribution of foodborne and waterborne illnesses to comprehensive transmission pathways. [file 20-0316-Techapp-s4.pdf]

Article DOI: https://doi.org/10.3201/eid2701.200316

Attribution of Illnesses Transmitted by Food and Water to Comprehensive Transmission Pathways Using Structured Expert Judgment, United States

Appendix 4

Knowledge Review Questionnaire and Results

Appendix 4. Review of Knowledge

Review of Knowledge

Transmission pathways & definitions

Please feel free to refer to the pathway definitions as needed to complete this. There are 20 questions in total.

* Required

1. **Email address** *

2. **First Name** *

3. **Last Name** *

Transmission Pathway Questions

Please choose the transmission pathway that best fits each scenario described

1. **Norovirus illness among attendees of a banquet linked to carpet and indoor environment that had been contaminated with vomit the day before the banquet and subsequently cleaned**

   Mark only one oval.

   - Foodborne transmission
   - Foodborne transmission - Food-handler related
   - Waterborne transmission - Drinking water
   - Waterborne transmission - Recreational water
   - Waterborne transmission - Non-recreational/Non-drinking
   - Person-to-person transmission
   - Animal contact transmission
   - Environmental transmission
   - Environmental- Presumed person-to-person
   - Environmental - Presumed animal contact

2. **Salmonellosis among participants in a mud volleyball tournament linked to ingestion of mud**
Mark only one oval.

- ◯ Foodborne transmission
- ◯ Foodborne transmission - Food-handler related
- ◯ Waterborne transmission - Drinking water
- ◯ Waterborne transmission - Recreational water
- ◯ Waterborne transmission - Non-recreational/Non-drinking
- ◯ Person-to-person transmission
- ◯ Animal contact transmission
- ◯ Environmental transmission
- ◯ Environmental- Presumed person-to-person
- ◯ Environmental - Presumed animal contact

3. **Norovirus illness from a lake after someone vomited in the lake**
Mark only one oval.

- ◯ Foodborne transmission
- ◯ Foodborne transmission - Food-handler related
- ◯ Waterborne transmission - Drinking water
- ◯ Waterborne transmission - Recreational water
- ◯ Waterborne transmission - Non-recreational/Non-drinking
- ◯ Person-to-person transmission
- ◯ Animal contact transmission
- ◯ Environmental transmission
- ◯ Environmental- Presumed person-to-person
- ◯ Environmental - Presumed animal contact

4. **STEC O157 illness linked to touching the railings of an animal enclosure at an animal fair**
Mark only one oval.

- ◯ Foodborne transmission
- ◯ Foodborne transmission - Food-handler related
- ◯ Waterborne transmission - Drinking water
- ◯ Waterborne transmission - Recreational water
- ◯ Waterborne transmission - Non-recreational/Non-drinking
- ◯ Person-to-person transmission
- ◯ Animal contact transmission
- ◯ Environmental transmission
- ◯ Environmental- Presumed person-to-person
- ◯ Environmental - Presumed animal contact

5. **Campylobacteriosis among mountain bikers linked to ingestion of mud**
Mark only one oval.

- ◯ Foodborne transmission
- ◯ Foodborne transmission - Food-handler related
- ◯ Waterborne transmission - Drinking water
- ◯ Waterborne transmission - Recreational water
- ◯ Waterborne transmission - Non-recreational/Non-drinking
- ◯ Person-to-person transmission
- ◯ Animal contact transmission
- ◯ Environmental transmission
- ◯ Environmental- Presumed person-to-person
- ◯ Environmental - Presumed animal contact

6. **STEC O157 illness linked to camping on grounds that had been used as pasture area for sheep 1 month prior**
Mark only one oval.

- ◯ Foodborne transmission
- ◯ Foodborne transmission - Food-handler related
- ◯ Waterborne transmission - Drinking water
- ◯ Waterborne transmission - Recreational water
- ◯ Waterborne transmission - Non-recreational/Non-drinking
- ◯ Person-to-person transmission
- ◯ Animal contact transmission
- ◯ Environmental transmission
- ◯ Environmental- Presumed person-to-person
- ◯ Environmental - Presumed animal contact

7. **Legionellosis linked to construction activities with a water main break**
Mark only one oval.

- ◯ Foodborne transmission
- ◯ Foodborne transmission - Food-handler related
- ◯ Waterborne transmission - Drinking water
- ◯ Waterborne transmission - Recreational water
- ◯ Waterborne transmission - Non-recreational/Non-drinking
- ◯ Person-to-person transmission
- ◯ Animal contact transmission
- ◯ Environmental transmission
- ◯ Environmental- Presumed person-to-person
- ◯ Environmental - Presumed animal contact

8. **Q-fever associated with living within 3 miles of an infected goat farm**
Mark only one oval.

- Foodborne transmission
- Foodborne transmission - Food-handler related
- Waterborne transmission - Drinking water
- Waterborne transmission - Recreational water
- Waterborne transmission - Non-recreational/Non-drinking
- Person-to-person transmission
- Animal contact transmission
- Environmental transmission
- Environmental- Presumed person-to-person
- Environmental - Presumed animal contact

9. **Brucellosis acquired through wounds or inhalation among employees at a pig slaughter plant**
Mark only one oval.

- Foodborne transmission
- Foodborne transmission - Food-handler related
- Waterborne transmission - Drinking water
- Waterborne transmission - Recreational water
- Waterborne transmission - Non-recreational/Non-drinking
- Person-to-person transmission
- Animal contact transmission
- Environmental transmission
- Environmental- Presumed person-to-person
- Environmental - Presumed animal contact

10. **Mycobacterium kansasii infection among mineworkers linked to contaminated showers**
Mark only one oval.

- Foodborne transmission
- Foodborne transmission - Food-handler related
- Waterborne transmission - Drinking water
- Waterborne transmission - Recreational water
- Waterborne transmission - Non-recreational/Non-drinking
- Person-to-person transmission
- Animal contact transmission
- Environmental transmission
- Environmental- Presumed person-to-person
- Environmental - Presumed animal contact

11. **Toxoplasmosis linked to working in the home garden**
Mark only one oval.

- ◯ Foodborne transmission
- ◯ Foodborne transmission - Food-handler related
- ◯ Waterborne transmission - Drinking water
- ◯ Waterborne transmission - Recreational water
- ◯ Waterborne transmission - Non-recreational/Non-drinking
- ◯ Person-to-person transmission
- ◯ Animal contact transmission
- ◯ Environmental transmission
- ◯ Environmental- Presumed person-to-person
- ◯ Environmental - Presumed animal contact

12. **Campylobacteriosis linked to contact with contaminated packaging of chicken meat**
Mark only one oval.

- ◯ Foodborne transmission
- ◯ Foodborne transmission - Food-handler related
- ◯ Waterborne transmission - Drinking water
- ◯ Waterborne transmission - Recreational water
- ◯ Waterborne transmission - Non-recreational/Non-drinking
- ◯ Person-to-person transmission
- ◯ Animal contact transmission
- ◯ Environmental transmission
- ◯ Environmental- Presumed person-to-person
- ◯ Environmental - Presumed animal contact

13. **Legionellosis linked to a contaminated cooling tower**
Mark only one oval.

- ◯ Foodborne transmission
- ◯ Foodborne transmission - Food-handler related
- ◯ Waterborne transmission - Drinking water
- ◯ Waterborne transmission - Recreational water
- ◯ Waterborne transmission - Non-recreational/Non-drinking
- ◯ Person-to-person transmission
- ◯ Animal contact transmission
- ◯ Environmental transmission
- ◯ Environmental- Presumed person-to-person
- ◯ Environmental - Presumed animal contact

14. **Salmonella in the family of a laboratorian who routinely prepared the family's dinners**
Mark only one oval.

- ◯ Foodborne transmission
- ◯ Foodborne transmission - Food-handler related
- ◯ Waterborne transmission - Drinking water
- ◯ Waterborne transmission - Recreational water
- ◯ Waterborne transmission - Non-recreational/Non-drinking
- ◯ Person-to-person transmission
- ◯ Animal contact transmission
- ◯ Environmental transmission
- ◯ Environmental- Presumed person-to-person
- ◯ Environmental - Presumed animal contact

15. **Transmission of STEC O157 illness from a sick child to other children in a daycare**
Mark only one oval.

- ◯ Foodborne transmission
- ◯ Foodborne transmission - Food-handler related
- ◯ Waterborne transmission - Drinking water
- ◯ Waterborne transmission - Recreational water
- ◯ Waterborne transmission - Non-recreational/Non-drinking
- ◯ Person-to-person transmission
- ◯ Animal contact transmission
- ◯ Environmental transmission
- ◯ Environmental- Presumed person-to-person
- ◯ Environmental - Presumed animal contact

16. **Salmonellosis due to internalization of contaminated water by tomatoes during processing**
Mark only one oval.

- ◯ Foodborne transmission
- ◯ Foodborne transmission - Food-handler related
- ◯ Waterborne transmission - Drinking water
- ◯ Waterborne transmission - Recreational water
- ◯ Waterborne transmission - Non-recreational/Non-drinking
- ◯ Person-to-person transmission
- ◯ Animal contact transmission
- ◯ Environmental transmission
- ◯ Environmental- Presumed person-to-person
- ◯ Environmental - Presumed animal contact

17. **Salmonellosis linked to rodents contaminating food in a restaurant kitchen**
Mark only one oval.

- ◯ Foodborne transmission
- ◯ Foodborne transmission - Food-handler related
- ◯ Waterborne transmission - Drinking water
- ◯ Waterborne transmission - Recreational water
- ◯ Waterborne transmission - Non-recreational/Non-drinking
- ◯ Person-to-person transmission
- ◯ Animal contact transmission
- ◯ Environmental transmission
- ◯ Environmental- Presumed person-to-person
- ◯ Environmental - Presumed animal contact

18.. **Norovirus outbreak due to an infected food handler preparing sandwiches**
Mark only one oval.

- ◯ Foodborne transmission
- ◯ Foodborne transmission - Food-handler related
- ◯ Waterborne transmission - Drinking water
- ◯ Waterborne transmission - Recreational water
- ◯ Waterborne transmission - Non-recreational/Non-drinking
- ◯ Person-to-person transmission
- ◯ Animal contact transmission
- ◯ Environmental transmission
- ◯ Environmental- Presumed person-to-person
- ◯ Environmental - Presumed animal contact

19. **Paratyphi B var. Java linked to contact with aquariums housing fish**
Mark only one oval.

- ◯ Foodborne transmission
- ◯ Foodborne transmission - Food-handler related
- ◯ Waterborne transmission - Drinking water
- ◯ Waterborne transmission - Recreational water
- ◯ Waterborne transmission - Non-recreational/Non-drinking
- ◯ Person-to-person transmission
- ◯ Animal contact transmission
- ◯ Environmental transmission
- ◯ Environmental- Presumed person-to-person
- ◯ Environmental - Presumed animal contact

20. **STEC O157 infection after attending a dance in a barn that had been cleaned since housing animals**

Mark only one oval.

- ◯ Foodborne transmission
- ◯ Foodborne transmission - Food-handler related
- ◯ Waterborne transmission - Drinking water
- ◯ Waterborne transmission - Recreational water
- ◯ Waterborne transmission - Non-recreational/Non-drinking
- ◯ Person-to-person transmission
- ◯ Animal contact transmission
- ◯ Environmental transmission
- ◯ Environmental- Presumed person-to-person
- ◯ Environmental - Presumed animal contact

Appendix 4 Figure 1. Knowledge Review Questionnaire

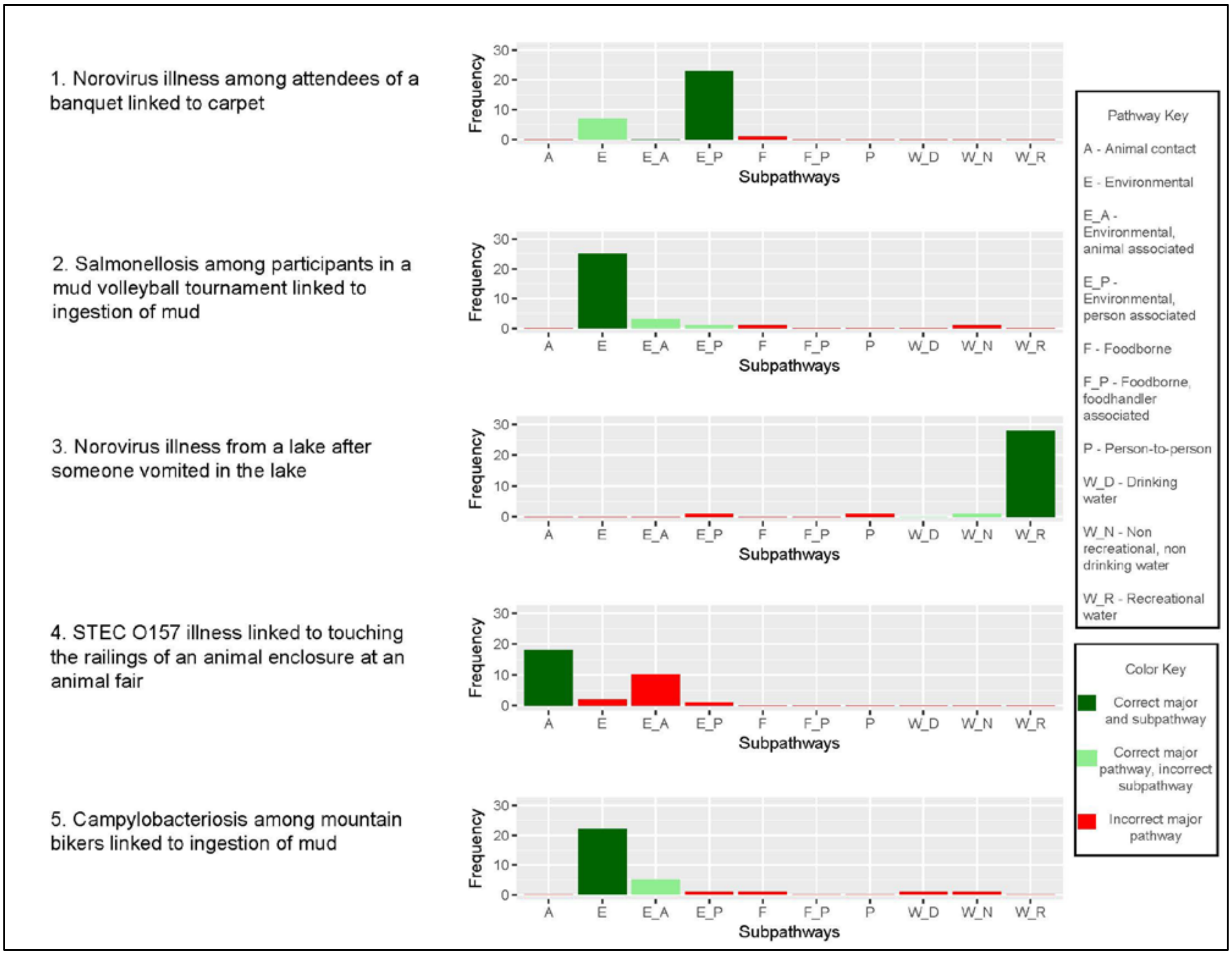
1. Norovirus illness among attendees of a banquet linked to carpet
2. Salmonellosis among participants in a mud volleyball tournament linked to ingestion of mud
3. Norovirus illness from a lake after someone vomited in the lake
4. STEC O157 illness linked to touching the railings of an animal enclosure at an animal fair
5. Campylobacteriosis among mountain bikers linked to ingestion of mud
Frequency
Subpathways
A E E_A E_P F F_P P W_D W_N W_R
Pathway Key
A - Animal contact
E - Environmental
E_A - Environmental, animal associated
E_P - Environmental, person associated
F - Foodborne
F_P - Foodborne, foodhandler associated
P - Person-to-person
W_D - Drinking water
W_N - Non recreational, non drinking water
W_R - Recreational water
Color Key
Correct major and subpathway
Correct major pathway, incorrect subpathway
Incorrect major pathway

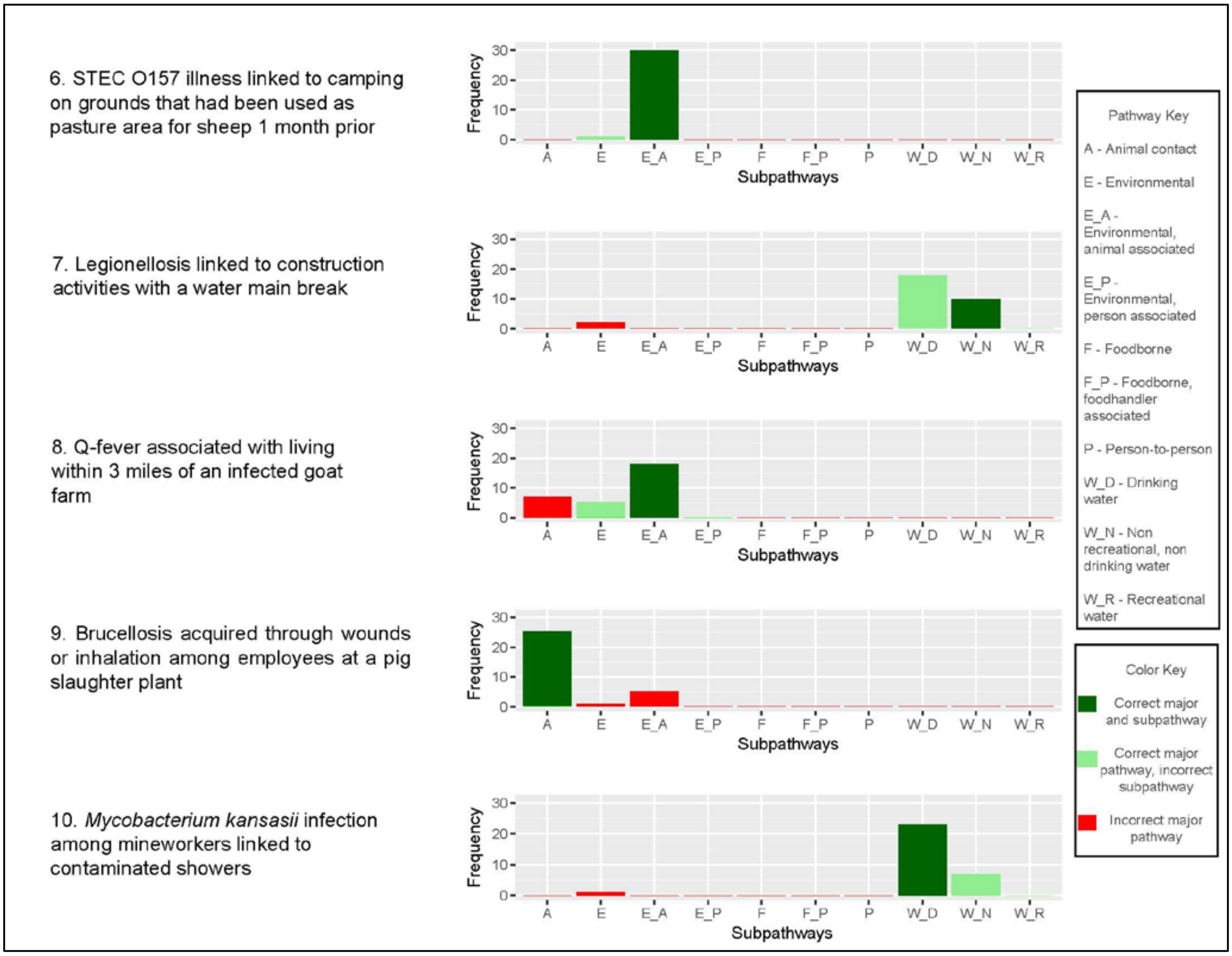
6. STEC O157 illness linked to camping on grounds that had been used as pasture area for sheep 1 month prior
7. Legionellosis linked to construction activities with a water main break
8. Q-fever associated with living within 3 miles of an infected goat farm
9. Brucellosis acquired through wounds or inhalation among employees at a pig slaughter plant
10. *Mycobacterium kansasii* infection among mineworkers linked to contaminated showers
Frequency
Subpathways
A
E
E_A
E_P
F
F_P
P
W_D
W_N
W_R
Pathway Key
A - Animal contact
E - Environmental
E_A - Environmental, animal associated
E_P - Environmental, person associated
F - Foodborne
F_P - Foodborne, foodhandler associated
P - Person-to-person
W_D - Drinking water
W_N - Non recreational, non drinking water
W_R - Recreational water
Color Key
Correct major and subpathway
Correct major pathway, incorrect subpathway
Incorrect major pathway

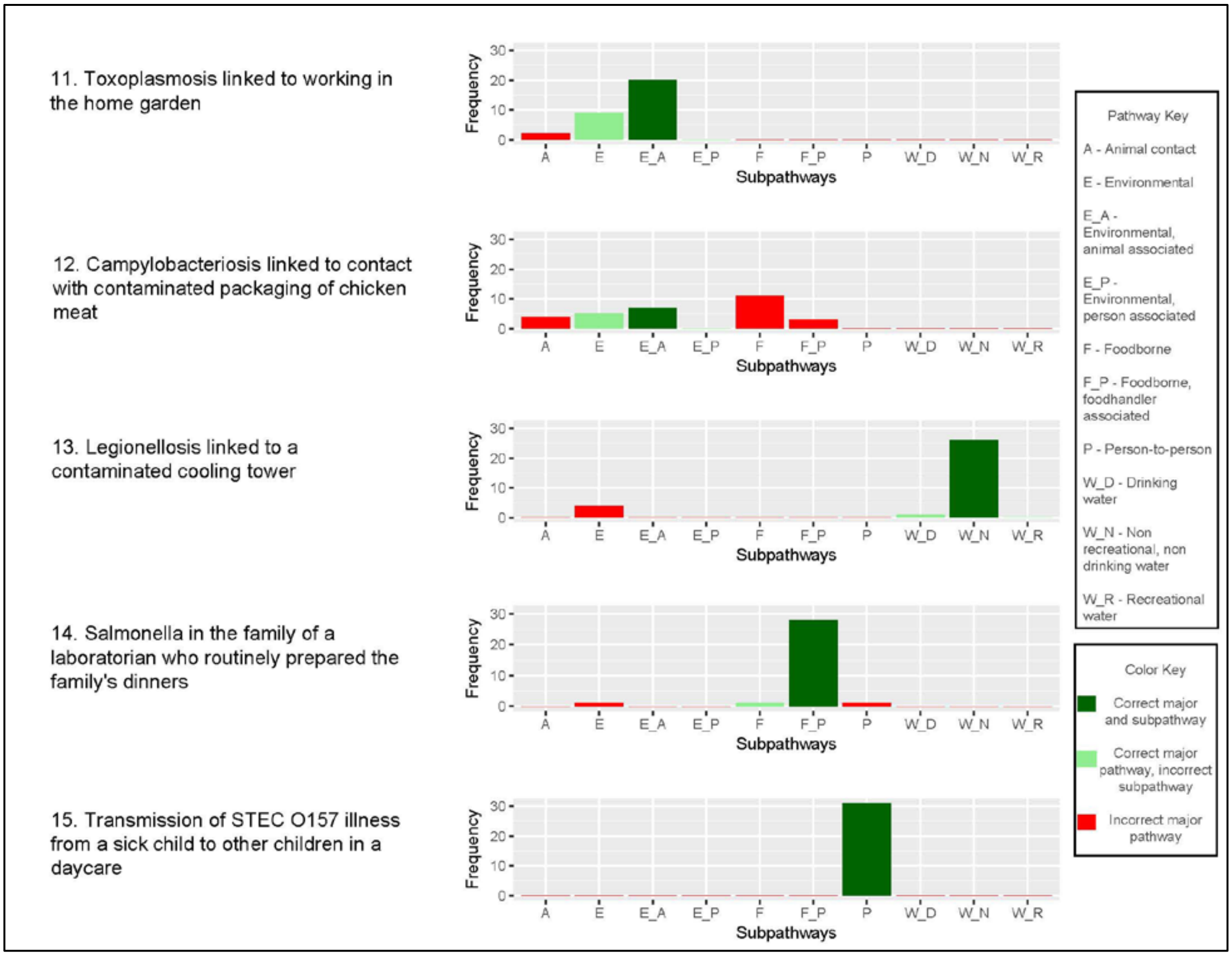
11. Toxoplasmosis linked to working in the home garden
12. Campylobacteriosis linked to contact with contaminated packaging of chicken meat
13. Legionellosis linked to a contaminated cooling tower
14. Salmonella in the family of a laboratorian who routinely prepared the family's dinners
15. Transmission of STEC O157 illness from a sick child to other children in a daycare
Frequency
0
10
20
30
A
E
E_A
E_P
F
F_P
P
W_D
W_N
W_R
Subpathways
Pathway Key
A - Animal contact
E - Environmental
E_A - Environmental, animal associated
E_P - Environmental, person associated
F - Foodborne
F_P - Foodborne, foodhandler associated
P - Person-to-person
W_D - Drinking water
W_N - Non recreational, non drinking water
W_R - Recreational water
Color Key
Correct major and subpathway
Correct major pathway, incorrect subpathway
Incorrect major pathway

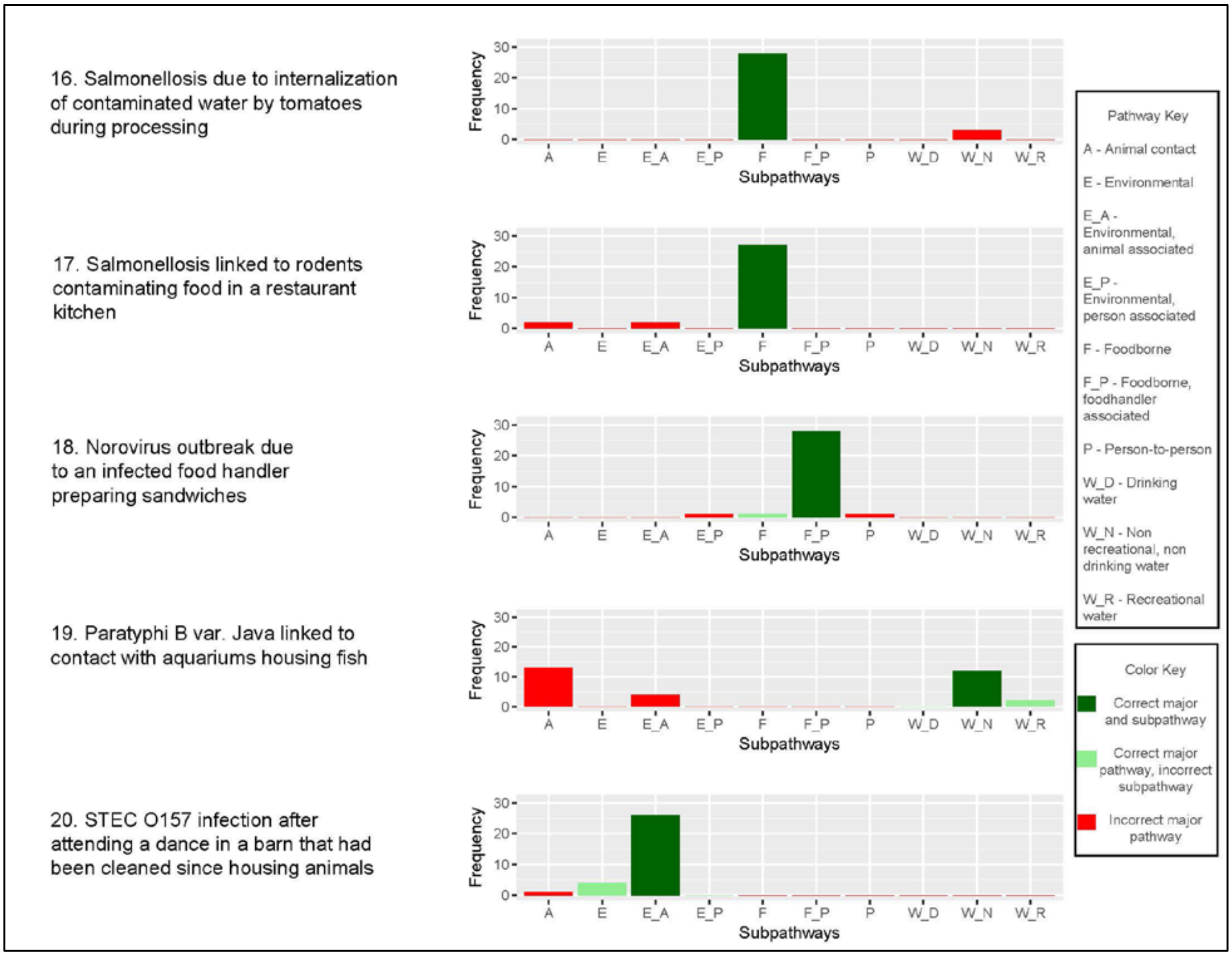

Appendix 4 Figure 2. Expert Responses to Knowledge Review Questionnaire
