# Supplementary material for: Attribution of Illnesses Transmitted by Food and Water to Comprehensive Transmission Pathways Using Structured Expert Judgment, United States
Source: Emerg Infect Dis. 2021 Jan;27(1):182–95. doi: 10.3201/eid2701.200316 (PMC7774530; doi:10.3201/eid2701.200316)
Supplement: Appendix 5 — Additional discussion about the validation analysis for structured expert judgment for attribution of foodborne and waterborne illnesses to comprehensive transmission pathways. [file 20-0316-Techapp-s5.pdf]

Article DOI: https://doi.org/10.3201/eid2701.200316

Attribution of Illnesses Transmitted by Food and Water to Comprehensive Transmission Pathways Using Structured Expert Judgment, United States

Appendix 5

Detailed Validation Analysis

This appendix focuses on validation results for a typical panel: Panel 6, involving 21 experts. The elicitation of Panel 6 included 14 calibration questions (or variables) and 11 target questions. Experts' assessments for calibration variables were evaluated in terms of statistical accuracy and informativeness. As always, statistical accuracy is the p value at which we would falsely reject the hypothesis that an expert's probabilistic assessments were statistically accurate. Informativeness reflects the degree to which an expert's distribution was concentrated, and was measured as relative information in relation to a background measure. For all cases presented here, the background measure was uniform. Relative information of distribution A with respect to distribution B reflects the surprise we should feel if we initially believed B and drew samples exhibiting distribution A. It is related to the log likelihood ratio commonly used in goodness of fit testing. The informativeness of an expert is computed as the average over the informativeness in the calibration variables. The informativeness of an expert can also be computed for all the questions, thus including the questions of interest.

A combined score was obtained by multiplying the statistical accuracy by the informativeness, which in turn, provided performance-based weights for the experts. The weighted combination of experts is referred to as the performance weighted decision maker (PWDM). We evaluated the PWDM as compared with the equally weighted decision maker (EWDM), which assigns equal weight to all experts. Any DM can be regarded as an expert itself; thus, its assessments can also be evaluated in terms of statistical accuracy and informativeness.

Intuitive definitions of the relevant terms are offered here; for precise mathematical definitions and detailed descriptions, the reader is referred to Colson and Cooke 2017 and 2018 (*1*,*2*), especially the supplementary online material.

The Classical Model for Structured Expert Judgment admits 3 types of validation approaches: robustness analysis, in-sample validation, and out-of-sample validation.

Panel 6 In-Sample Validation

In-sample validation considers the statistical accuracy (p value) and informativeness of PWDM and EWDM, evaluated with respect to all 14 calibration variables. From Appendix 5 Table 1 we see that the statistical accuracy scores of the experts range from 0.57 (expert 3) to 0.00000064 (expert 10). Intuitively, this means that if we reject the hypothesis that expert 3 is statistically accurate, we have a 57% chance of being wrong, whereas with expert 10, the chance of being wrong is 0.00000064. Informativeness is tabulated for all variables (calibration and variables of interest combined), as well as for the calibration variables only.

As mentioned earlier, an expert's combined score is computed as the product of the p value (statistical accuracy) and informativeness for calibration variables, which, in turn, leads to experts' weights. The experts' weights can be calculated when taking into account all calibration questions, but can also be calculated for each calibration question separately. We refer to this case as item weights; experts will receive a different weight for each question, which depends on their informativeness for each question.

The expert weights should satisfy an asymptotic "proper scoring rule" property; that is, an expert maximizes his or her expected weight in the long run by, and only by, giving assessments corresponding to his or her true beliefs. Performance weights are asymptotic strictly proper scoring rules if there is some positive value α such that an expert is unweighted if his or her p value falls below α. The optimal performance weighted DM is computed by finding an optimal α cutoff for p values, which is chosen to maximize the combined score of the resulting PW. (In this exercise, PW means the item-specific PW where weights for each variable are inflected with the expert's information score for that variable.) For Panel 6, the optimal cutoff value was 0.2426, resulting in 5 experts being weighted (in bold in Appendix 5 Table 1). The expert and DM scores are given in Appendix 5 Table 1.

In-sample validation consists of ascertaining that the statistical accuracy of the PWDM and EWDM is acceptable without sacrificing informativeness. This is termed “in-sample validation” because the PWDM’s performance is assessed on the same set of calibration variables that were used to initialize the PWDM. From Appendix 5 Table 1 we see that PWDM is more statistically accurate than EWDM, but that both are acceptable. PWDM’s informativeness is comparable to the lower values of the experts, whereas EWDM’s informativeness is well below that of the experts. This replicates a recurring finding that EWDM tends to purchase acceptable statistical performance at the expense of informativeness.

Robustness

Robustness analysis removes 1 expert or 1 calibration variable at a time and recomputes the PWDM. The statistical accuracy and informativeness of the “perturbed decision makers” are compared with the original statistical accuracy and informativeness and the “discrepancy” between the perturbed DM and the original DM is computed. Mathematically, this corresponds to the relative information of each expert’s distribution with respect to the PW combination. We compare this discrepancy with the discrepancy between each expert and the EWDM. The later discrepancy gives an indication of the disagreement among the experts themselves. When the latter discrepancies are much greater than the former, we may conclude that the PWDM is indeed robust: the change induced by loss of expert or loss of item is then small relative to the differences between the experts themselves. These discrepancies between each expert and EWDM are given in Appendix 5 Table 2, whereas the discrepancies relative to the original PWDM are given in Appendix 5 Table 3.

The average of these discrepancies gives an index for the disparity within the expert panel. The higher the expert’s discrepancy relative to EWDM, the higher the disagreement with the DM. Note that the discrepancy for all 5 weighted experts is below the average discrepancy over all experts. This indicates that the weighted experts among themselves show better agreement than the experts overall.

Appendix 5 Table 3 shows the results for robustness analysis on calibration variables. That is, each of the 14 calibration questions has been excluded, one at a time, from the analysis. The optimal performance-based DM, using item weights, for the remaining 13 calibration

variables is obtained and its resulting informativeness and p value are provided. Furthermore, the discrepancy is also reflected by the total relative information with respect to the original DM, based on the 14 calibration questions. The informativeness of the new DM varies between 0.93 and 1.62, and therefore does not change significantly when removing calibration variables. However, the p value increases significantly, to 0.92, when removing CAL022, CAL055, CAL088, CAL099, or CAL1111, in turn. Nonetheless, the average of the perturbed discrepancies is 0.269, which is much smaller than the discrepancy among the experts themselves in Appendix 5 Table 2 (0.807). The PWDM is therefore shown to be robust against the loss of a single calibration variable.

Appendix 5 Table 4 shows the results of robustness on experts. Similarly to the robustness on calibration variables, experts were excluded one at a time and the optimal PWDM, using item weights, was obtained for the remaining 20 experts. The informativeness and statistical accuracy, as well as discrepancy compared to the original PWDM, are provided. The statistical accuracy of the new DM is, except when excluding expert 48, the same as the initial DM's p value. Similarly, the informativeness accounts for small variations. Finally, the average discrepancy is 0.07, which indicates a very small discrepancy with respect to the original DM.

We may conclude that the PWDM results for Panel 6 are robust with respect to loss of a single calibration variable and are extremely robust relative to the loss of a single expert.

Out-of-Sample Validation

Out-of-sample validation requires that the PWDM and EWDM be scored on a different set of variables as those used to initialize the weighting model. Because we cannot observe the variables of interest, we must recourse to cross validation: every non-empty subset of calibration variables is used to initialize the model (usually referred to as the training set) and performance is scored using predictions of variables in the complementary set (usually referred to as the test set). With 14 calibration variables, this involves $2^{14} - 2 = 16,832$ training set/test set computations. This accounts for training sets of size varying from 1 to 13, which include all possible combinations of calibration variables. A small training set has low statistical power for resolving the experts' performance and thus produces combinations that are not representative of the final expert panel. On the other hand, a small test set has low statistical power for resolving

the performance of the PWDM and EWDM. As the test set size decreases, statistical accuracy is evaluated by tests of decreasing statistical power and all statistical accuracy scores tend to rise. It is argued that using 80% of the calibration variables in the training set is a good compromise (*1*). (These results are computed with the MATLAB code graciously provided by Lt. Col. Justin Eggstaff.) For the results presented here, the EWDM and global PWDM scores were averaged over all same-sized training sets.

Whereas Appendix 5 Table 1 used item-specific performance weighting, for out-of-sample validation, computational constraints impose global performance weighting: instead of weighting experts for each variable using the experts' information scores for the given variable, an expert's average information over all calibration variables is used to derive weights that apply to all variables. With item-specific weights, an expert can up- or downweight himself or herself variable-wise by choosing a more or less informative distribution for the given variable. Item-specific weighting usually outperforms global weighting, and this was true for Panel 6.

The out-of-sample scores for statistical accuracy averaged over same-sized training sets are shown in Appendix 5 Figure 1 panel A. There is an out-of-sample penalty for the statistical accuracy score, but this penalty is small in absolute terms. As the training set grows, the penalty shrinks, and the PWDM resembles the PWDM of original study based on all calibration variables. Out-of-sample informativeness of PWDM is consistently higher than that of EWDM (Appendix 5 Figure 1 panel B). Putting these two together in Appendix 5 Figure 2, the combined score of PWDM is clearly superior to that of EWDM out-of-sample. The advised training set sample size of 80% of all calibration variables is highlighted.

All Experts: In-Sample

Because all 48 experts assessed the same 14 calibration variables, it is also possible to consider a fictitious panel consisting of all 48 experts. Robustness analysis does not make sense, as the 48 experts did not assess the same variables of interest. However, in- and out-of-sample validation can be performed.

In Appendix 5 Table 5 the scores for all 48 experts are shown ranked according to their combined scores. The 15 best performing experts are highlighted (shaded yellow). The last 4 rows compare 4 different DMs. PWDM is the optimal performance item weighted DM. PWDM

minus 15 represents a mass extinction robustness analysis: the 15 top performing experts, which are shaded in yellow, are removed and PWDM is computed for the remaining experts. PWDMNoOpt uses all 48 experts but sets the cutoff at zero; all experts are weighted with weights proportional to their combined score. EWDM is the equal weighted combination of all 48 experts. Experts' information scores in Appendix 5 Table 5 are higher than those in Appendix 5 Table 1 because informativeness is scored relative to the uniform distribution spanning all assessments of all experts. Increasing the number of experts expands the range of this uniform distribution, making all experts appear more informative.

PWDM minus 15 scores better than PWDMNoOpt and better than EWDM. This shows the robustness of the classical model under massive expert loss: removing the top performing third of the experts still produces higher performance scores than equally weighting all experts. The role of optimization is also highlighted. If optimization is not performed, the result PWDMNoOpt is only marginally better than EWDM.

All Experts: Out-of-Sample

The explanations given for Panel 6 apply here as well. Appendix 5 Figures 3 and 4 correspond to Appendix 5 Figures 1 and 2.

Conclusion

This appendix illustrates the 3 types of validation that are available within the Classical Model for Structured Expert Judgment: robustness analysis, in-sample validation, and out of-sample validation. With regard to the data from the CDC study, we may conclude that all three types of validation are strongly attested.

References

1. Colson A, Cooke RM. Cross validation for the classical model of structured expert judgment. Reliab Eng Syst Saf. 2017;163:109–20. https://doi.org/10.1016/j.ress.2017.02.003

2. Colson A, Cooke RM. Expert elicitation: using the classical model to validate experts' judgments. Rev Environ Econ Policy. 2018;12:113–32. https://doi.org/10.1093/reep/rex022

Appendix 5 Table 1. Panel 6 performance scores of the 21 experts, the PWDM, and EWDM*

Expert	p value	Informativeness, all variables	Informativeness, calibration variables	Combined score
Expert 01	0.000720	2.394	1.894	0.001
Expert 04	0.0135	2.361	1.906	0.026
Expert 15	0.00984	2.12	2.169	0.021
Expert 18	0.000000738	3.662	3.221	0
Expert 29	0.0334	2.498	1.396	0.047
**Expert 33**	**0.243**	**2.331**	**1.468**	**0.356**
Expert 43	0.00126	2.751	1.923	0.002
**Expert 48**	**0.569**	**2.458**	**1.541**	**0.877**
**Expert 03**	**0.569**	**1.686**	**1.526**	**0.868**
**Expert 07**	**0.243**	**2.043**	**1.671**	**0.405**
Expert 10	0.000000638	1.21	1.07	0
Expert 17	0.144	2.327	1.613	0.231
Expert 24	0.00984	1.708	1.734	0.017
Expert 25	0.00984	2.377	1.416	0.014
Expert 27	0.000101	1.664	1.514	0
Expert 32	0.0543	1.869	1.353	0.073
**Expert 47**	**0.569**	**1.02**	**0.8906**	**0.507**
Expert 16	0.0724	1.821	1.502	0.109
Expert 42	0.223	2.284	2.114	0.47
Expert 06	0.185	2.186	2.177	0.403
Expert 22	0.00217	3.319	2.718	0.006
PWDM	0.659	1.473	1.093	0.72
EWDM	0.1325	0.8184	0.6998	0.093

*EWDM, equally weighted decision maker; PWDM, performance weighted decision maker.The experts included in the optimal DM are in bold.

Appendix 5 Table 2. Expert discrepancies for each expert in Panel 6 with respect to the EW combination of the experts' distributions

Expert	Discrepancy relative to EWDM,* all variables
Expert 01	1.472
Expert 04	1.189
Expert 15	1.013
Expert 18	2.19
Expert 29	0.947
Expert 33	0.835
Expert 43	0.986
Expert 48	0.803
Expert 03	0.699
Expert 07	0.854
Expert 10	0.815
Expert 17	0.837
Expert 24	1.117
Expert 25	1.017
Expert 27	1.07
Expert 32	0.949
Expert 47	0.664
Expert 16	0.747
Expert 42	1.084
Expert 06	1.36
Expert 22	1.474
Average	1.003

*EWDM, equally weighted decision maker.

Appendix 5 Table 3. Robustness on calibration variables

Excluded variable	Informativeness calibration variables	p value	Discrepancy with respect to original decision maker (DM) calibration variables
CAL011	1.37	0.6894	0.2476
CAL022	1.134	0.9281	0.2195
CAL033	1.126	0.614	0.1117
CAL044	1.094	0.4209	0.171
CAL055	0.928	0.9281	0.2293
CAL066	0.919	0.614	0.06939
CAL077	1.309	0.614	0.2772
CAL088	1.62	0.9281	0.4339
CAL099	1.142	0.9281	0.2263
CAL1010	1.149	0.614	0.093
CAL1111	0.951	0.9281	0.4727
CAL1212	1.522	0.5285	0.4966
CAL1313	1.217	0.6894	0.1641
CAL1414	1.621	0.5285	0.5567
Original	1.093	0.659	
Average discrepancy			0.269

Appendix 5 Table 4. Robustness on experts

Excluded expert	Informativeness calibration variables	p value	Discrepancy with respect to original PWDM,* all variables
Expert 01	1.093	0.659	0.000000127
Expert 04	1.093	0.659	0.0000000653
Expert 15	1.093	0.659	0.0000000273
Expert 18	1.093	0.659	0.00233
Expert 29	1.093	0.659	0.0000000203
Expert 33	1.114	0.659	0.102
Expert 43	1.093	0.659	0.0000000189
Expert 48	1.076	0.968	0.485
Expert 03	1.074	0.659	0.179
Expert 07	1.083	0.659	0.182
Expert 10	0.665	0.659	0.018
Expert 17	1.093	0.659	0.0000000348
Expert 24	1.08	0.659	0.000361
Expert 25	1.092	0.659	0.000289
Expert 27	1.093	0.659	0.0000000243
Expert 32	1.093	0.659	0.00746
Expert 47	1.319	0.659	0.479
Expert 16	1.089	0.659	0.012
Expert 42	1.103	0.659	0.09
Expert 06	1.093	0.659	0.000000121
Expert 22	1.093	0.659	0.00000124
None	1.093	0.659	
Average discrepancy			0.0744

*PWDM, performance weighted decision maker.

Appendix 5 Table 5. All experts statistical accuracy (p value), informativeness, and combined scores*

Expert	p value	Informativeness calibration variables	Combined score
Expert013	0.968	2.54	2.46
Expert019	0.569	2.57	1.47
Expert041	0.569	2.28	1.30
Expert048	0.569	2.27	1.29
Expert003	0.569	2.26	1.28
Expert050	0.569	1.72	0.981
Expert047	0.569	1.59	0.906
Expert028	0.321	2.19	0.701
Expert042	0.223	2.85	0.633
Expert007	0.243	2.39	0.580
Expert006	0.185	2.91	0.540
Expert033	0.243	2.20	0.533
Expert049	0.223	2.19	0.487
Expert035	0.144	2.65	0.380
Expert017	0.144	2.34	0.336
Expert030	0.0909	2.91	0.264
Expert005	0.0909	2.69	0.244
Expert026	0.0909	2.22	0.201
Expert039	0.0724	2.55	0.185
Expert016	0.0724	2.21	0.160
Expert012	0.0483	2.44	0.118
Expert040	0.0483	2.41	0.116
Expert032	0.0543	2.08	0.113
Expert014	0.0339	2.47	0.0836
Expert021	0.0334	2.46	0.0820
Expert029	0.0334	2.12	0.0709
Expert004	0.0135	2.64	0.0355
Expert020	0.0124	2.73	0.0340
Expert044	0.00984	2.92	0.0287
Expert015	0.00984	2.90	0.0285
Expert002	0.00984	2.59	0.0255
Expert045	0.0119	2.04	0.0243
Expert024	0.00984	2.47	0.0243
Expert025	0.00984	2.14	0.0211
Expert011	0.00678	2.93	0.0199
Expert022	0.00217	3.45	0.00748
Expert034	0.00220	2.60	0.00573
Expert043	0.00126	2.66	0.00335
Expert001	0.000720	2.63	0.00189
Expert037	0.000276	2.53	0.000696
Expert009	0.000157	2.54	0.000398
Expert027	0.000101	2.24	0.000228
Expert036	0.0000190	3.66	0.0000698
Expert046	0.0000123	2.30	0.0000283
Expert008	0.00000211	3.38	0.00000713
Expert018	0.000000738	3.96	0.00000292
Expert023	0.000000580	2.07	0.00000120
Expert010	0.000000638	1.80	0.00000115
PWDM	0.968	2.54	2.46
PWDM minus 15	0.659	1.97	1.30
PWDMNoOpt	0.250	1.42	0.356
EWDM	0.250	1.08	0.270

*EWDM, equally weighted decision maker; PWDM, performance weighted decision maker. PWDM is optimal performance weighted DM, using item weights. PWDM minus 15 is the result of removing the 15 experts with best statistical accuracy, shaded yellow. PWDMNoOpt is a performance-based DM, with no optimization. For EWDM, each expert receives equal weight.

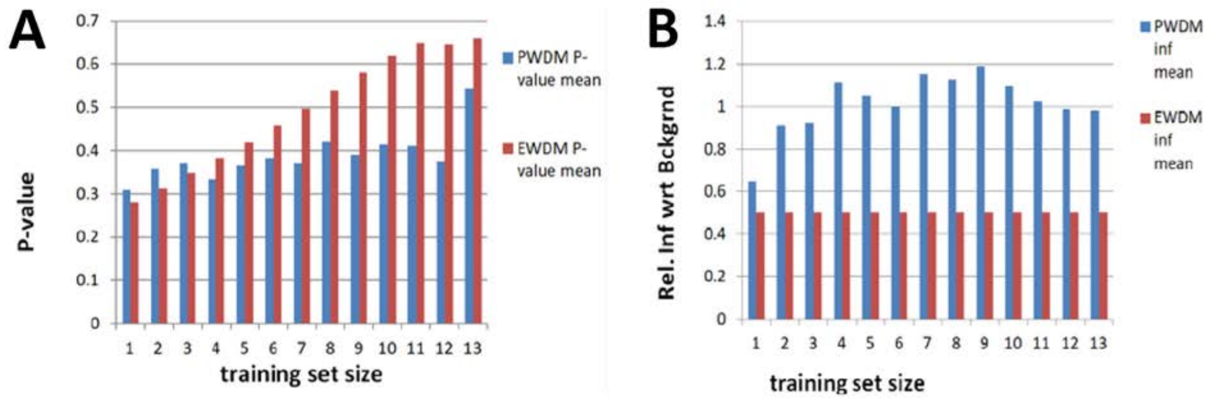

Appendix 5 Figure 1. A) Statistical accuracy and B) informativeness scores out of sample. EWDM, equally weighted decision maker; PWDM, performance weighted decision maker.

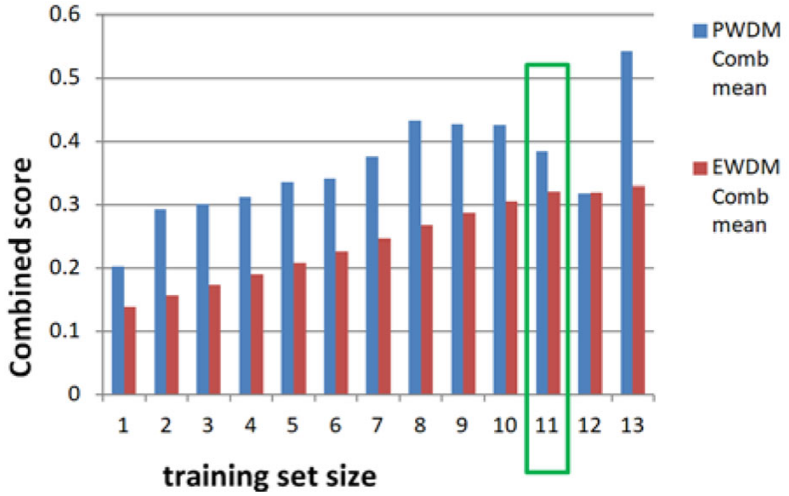

Appendix 5 Figure 2. Combined scores out of sample. Score for training set at 80% of calibration variables is highlighted. EWDM, equally weighted decision maker; PWDM, performance weighted decision maker.

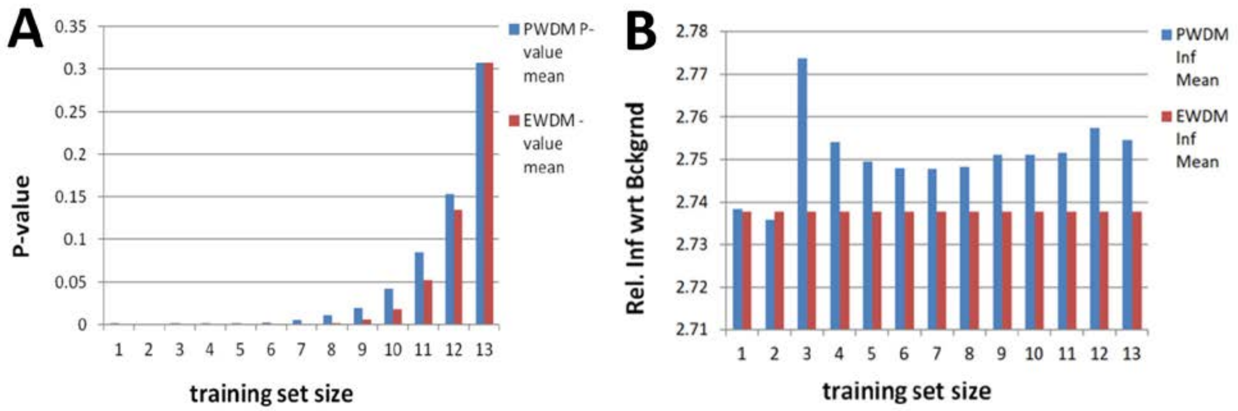

Appendix 5 Figure 3. All experts, A) statistical accuracy and B) information scores out of sample. EWDM, equally weighted decision maker; PWDM, performance weighted decision maker.

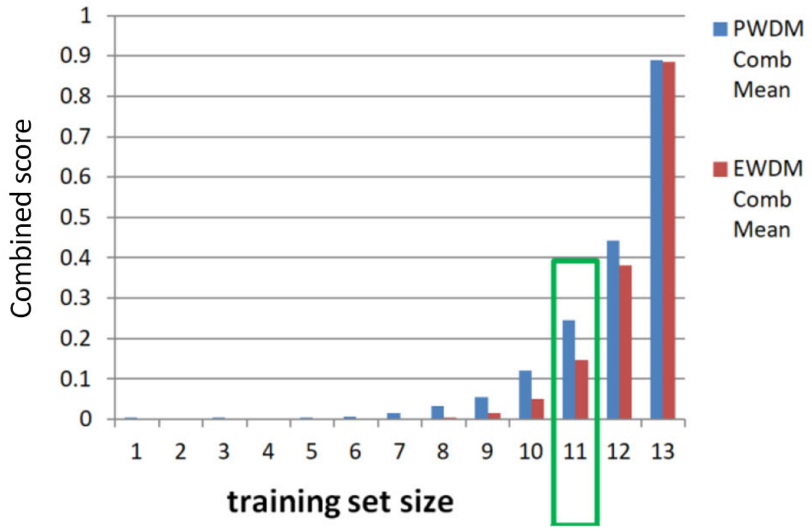

Appendix 5 Figure 4. All experts combined scores out of sample. Score for training set at 80% of calibration variables is highlighted. EWDM, equally weighted decision maker; PWDM, performance weighted decision maker.
